# DNA Methylomes Reveal Biological Networks Involved in Human Eye Development, Functions and Associated Disorders

**DOI:** 10.1038/s41598-017-12084-1

**Published:** 2017-09-18

**Authors:** María Berdasco, Antonio Gómez, Marcos J. Rubio, Jaume Català-Mora, Vicente Zanón-Moreno, Miguel Lopez, Cristina Hernández, Shigeo Yoshida, Takahito Nakama, Keijiro Ishikawa, Tatsuro Ishibashi, Amina M. Boubekeur, Lotfi Louhibi, Miguel A Pujana, Sergi Sayols, Fernando Setien, Dolores Corella, Carmen de Torres, Andreu Parareda, Jaume Mora, Ling Zhao, Kang Zhang, Matilde E. Lleonart, Javier Alonso, Rafael Simó, Josep M. Caminal, Manel Esteller

**Affiliations:** 1 Cancer Epigenetics Group; Cancer Epigenetics and Biology Program (PEBC), Bellvitge Biomedical Research Institute (IDIBELL), Barcelona, Catalonia, Spain; 2 Department of Ophthalmology, Bellvitge University Hospital, L’Hospitalet de Llobregat, Catalonia, Spain; 3 Hospital Sant Joan de Deu, Esplugues de Llobregat, Barcelona, Catalonia Spain; 4 Unidad de Epidemiología Molecular y Genética del Departamento de Medicina Preventiva y Salud Pública de la Facultad de Medicina y Odontología y Centro de Investigación en Red Fisiopatología de la Obesidad y Nutrición (CIBERobn), Universidad de Valencia, Valencia, Spain; 5 Unidad de Tumores Sólidos Infantiles, Instituto de Investigación de Enfermedades Raras, Instituto de Salud Carlos III, Majadahonda, Madrid, Spain; 6 Diabetes and Metabolism Research Unit, Vall d’Hebron Research Institute, Universitat Autònoma de Barcelona, Catalonia, Spain; 7 Department of Ophthalmology, Kyushu University Graduate School of Medical Sciences, Fukuoka, Japan; 8 Faculty of Life and Natural Science, Department of Applied Molecular Genetics, Laboratory of Medical Genetics, University of Science and Technology of Oran, Mohamed Boudiaf, Oran, Algeria; 9 Cancer Systems Biology Group; Program Against Cancer Therapeutic Resistance (ProCURE); Catalan Institute of Oncology (ICO), Bellvitge Biomedical Biomedical Research Institute (IDIBELL), Barcelona, Catalonia, Spain; 10 Shiley Eye Institute and Institute for Genomic Medicine, University of California, San Diego, La Jolla United States; 11 Biomedical Research in Cancer Stem Cells Group, Vall d’Hebron Research Institute (VHIR), Barcelona, Catalonia Spain; 12 Centro de Investigación Biomédica en Red de Diabetes y Enfermedades Metabólicas Asociadas (CIBERDEM), Instituto de Salud Carlos III (ISCIII), Madrid, Spain; 13 Department of Physiological Sciences II, School of Medicine, University of Barcelona, Barcelona, Catalonia Spain; 14 Institució Catalana de Recerca i Estudis Avançats (ICREA), Barcelona, Catalonia Spain

## Abstract

This work provides a comprehensive CpG methylation landscape of the different layers of the human eye that unveils the gene networks associated with their biological functions and how these are disrupted in common visual disorders. Herein, we firstly determined the role of CpG methylation in the regulation of ocular tissue-specification and described hypermethylation of retinal transcription factors (i.e., PAX6, RAX, SIX6) in a tissue-dependent manner. Second, we have characterized the DNA methylome of visual disorders linked to internal and external environmental factors. Main conclusions allow certifying that crucial pathways related to Wnt-MAPK signaling pathways or neuroinflammation are epigenetically controlled in the fibrotic disorders involved in retinal detachment, but results also reinforced the contribution of neurovascularization (ETS1, HES5, PRDM16) in diabetic retinopathy. Finally, we had studied the methylome in the most frequent intraocular tumors in adults and children (uveal melanoma and retinoblastoma, respectively). We observed that hypermethylation of tumor suppressor genes is a frequent event in ocular tumors, but also unmethylation is associated with tumorogenesis. Interestingly, unmethylation of the proto-oncogen RAB31 was a predictor of metastasis risk in uveal melanoma. Loss of methylation of the oncogenic mir-17-92 cluster was detected in primary tissues but also in blood from patients.

## Introduction

Charles Darwin wrote in “*The Origin of Species*” that the human eye was an organ of extreme perfection that pushed to the limit its theory about natural selection. In his own words: “*Reason tells me*, *that if numerous gradations from a simple and imperfect eye to one complex and perfect can be shown to exist*, *each grade being useful to its possessor*, *as is certainly the case; if further*, *the eye ever varies and the variations be inherited*, *as is likewise certainly the case; and if such variations should be useful to any animal under changing conditions of life*, *then the difficulty of believing that a perfect and complex eye could be formed by natural selection*, *though insuperable by our imagination*, *should not be considered as subversive of the theory*”. Its evolution represents a graduation from the simplest form (an optic nerve merely coated with pigment) to the most complex (the advanced optical systems of mammals). The layers of the eye, especially the retina, are highly complex and have frequently served as a model of central nervous system development^1^. Development and maintenance of the complexity of the human eye proceed by the fine-tuned regulation of gene expression in space and time. Apart from the regulation by the dynamic DNA-binding transcription factor, epigenetic factors may also be central to controlling changes in gene expression in the development of the eye^2–4^. Indeed, the role of epigenetics during eye development has been described in several experimental systems: reduction in the expression levels of histone deacetylase HDAC4, in the histone methyltransferase G9a, and in the miRNA processing enzyme Dicer during normal retinal development lead to apoptosis of retinal cells^5–7^. A dynamic expression pattern of DNA methyltransferase, Dnmt1 and Dnmt3, from immature to adult retina has also been reported^8^.

Aging-related eye diseases have become a priority problem in public health services due to their increasing prevalence in the general population and the seriousness of their impact on the quality of life of patients. For this reason, in recent years increased attention has been paid to the molecular biology of ocular diseases, especially the genetic basis of these disorders^9,10^. However, epigenetic studies are still in their infancy, and most of the research effort so far has focused on the role of epigenetics in regulating genes involved in retinal cell fate during embryogenesis, and the epigenetic alterations associated with tumoral disorders^2–4^. To date, macular degeneration is the only non-tumoral visual disorder in which CpG methylation has been explored^11^.

In this study we assessed the epigenetic characterization of visual disorders from a broader perspective, investigating the involvement of genome-wide CpG methylation in ocular diseases associated with environmental factors, inflammation and aging (diabetic retinopathy, proliferative vitreoretinopathy), and in ocular tumors (uveal melanoma and retinoblastoma). Characterizing the normal epigenetic patterns that govern the maintenance of tissue-specificity of the adult mature eye is as important as profiling alterations in pathologies. Considering both approaches (healthy *versus* pathological), we generated the largest CpG methylation map of embryonic and mature eye layers and their associated ocular diseases created so far. These data could be integrated to provide a comprehensive understanding of the epigenetic, genetic and environmental factors underlying visual disorders.

## Results

### DNA methylation signature in ocular tissue-types

**Figure 1:**
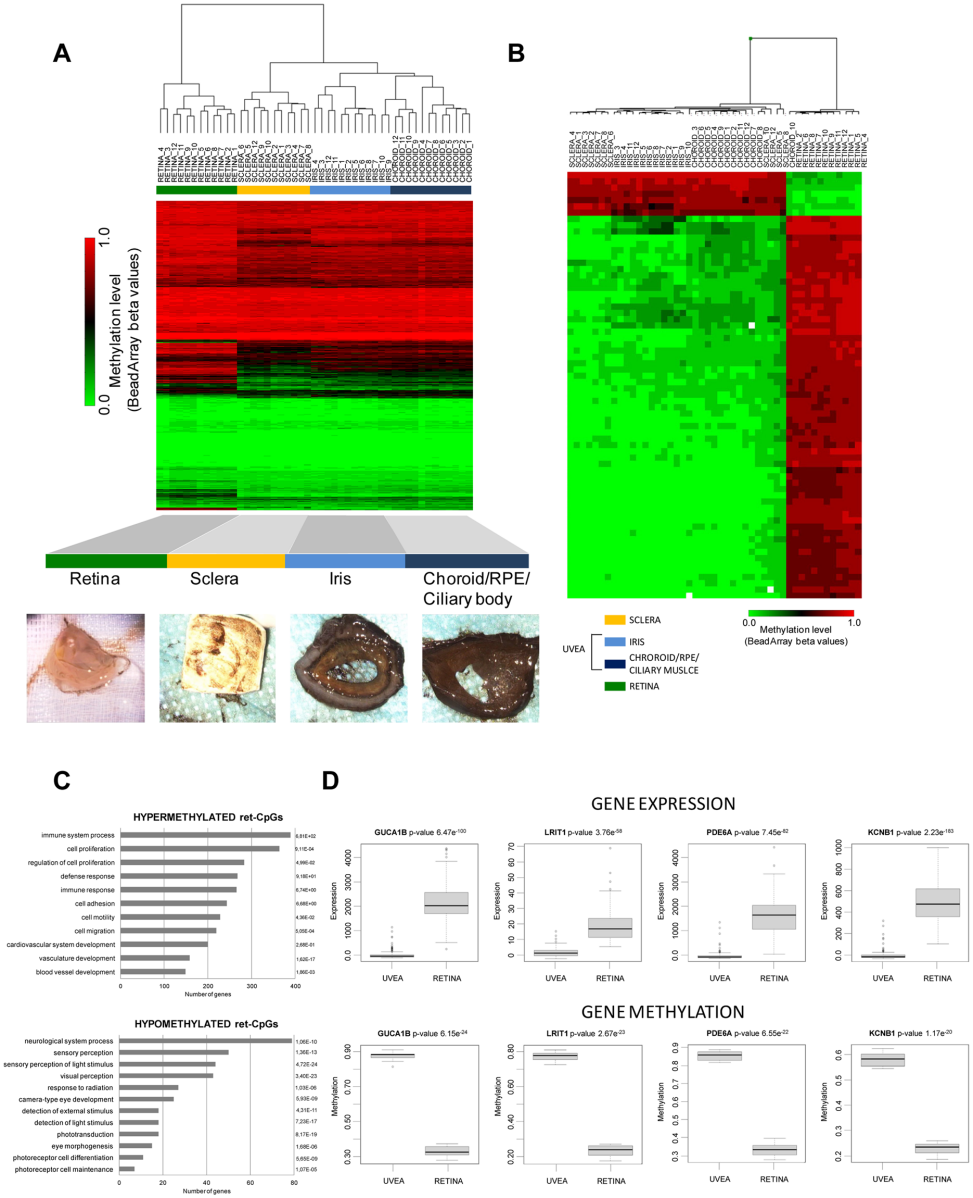
Comparative CpG methylation analysis of main ocular tissues. (**A**) Unsupervised heatmap clustering of β-values from CpG methylation array of the four eye tissues. Post-mortem whole eyes without ocular diseases were dissected into: (i) iris, (ii) choroid/RPE/ciliary body, (iii) sclera and (iv) retina. Red and green colors indicate high and low levels of DNA methylation, respectively. (**B**) Hierarchical clustering using the 24,683 significantly differentially methylated CpGs in the retina (ret-CpGs) and the remaining ocular tissues analyzed in the array. (**C**) Gene ontology analysis of genes associated with hypermethylated ret-CpGs (*above*) and hypomethylated ret-CpGs (*below*). The length of each bar is proportional to the number of differentially expressed genes in the functional category. Numbers on the right represent the statistically significant p-values associated with a one-sided Fisher’s exact test. (**D**) Correlation analysis of gene expression data from GEO Omnibus database GSE29801 (*above*) and CpG methylation value obtained from Infinium 450 K array (*below*) for four retina-specific hypomethylated genes (GUCA1B, LRIT1, PDE6A and KCNB1).

To determine whether DNA methylation is involved in the maintenance of tissue specification in the eye, we studied the CpG methylation profiles in the principal tissues of the human eye (n = 12 eyes from 6 donors) (Supplementary Table S1). Manual dissection of the eyes allows the isolation of the following tissue types: (*i*) iris, (*ii*) choroid/RPE/ciliary body, (*iii*) sclera and (*iv*) retina. The substantial involvement of CpG methylation in tissue commitment was reflected by the clear segregation of the ocular layers in an unsupervised cluster exclusively using the methylation signals of the CpGs contained in the arrays (Fig. 1A). Statistical analysis revealed that the CpG methylation in retina cells (with prominent neuronal compositions) clearly differs more from the other tissue types (of mainly mesenchymal composition) than the other tissues differed from each other, as reflected in the supervised cluster analysis (Fig. 1B). We found that the methylation level of 24,683 CpG sites significantly differ between the retina and the other tissue-types (termed “ret-CpGs”) (Supplementary Dataset 1). By contrast, fewer CpGs (n = 679) (Supplementary Dataset 1) differed between sclera cells and the uveal tract (defined as the average methylation values of iris plus choroid/RPE/ciliary body); the former is mainly composed of connective tissue that provides the structural framework of the eye, and the latter is highly vascularized and has a higher myocyte content than the sclera. Finally, only 25 CpGs differed (Supplementary Dataset 1) between the components of the uvea, the iris and choroid/RPE/ciliary body, consistent with the tissue similarity between them. The high degree of methylation found in the retina cells should be noted: 21,448 CpGs were hypermethylated in retina compared to all of the other three tissues (87%) while only 3,235 CpGs were hypomethylated (13%) (Supplementary Dataset 1; Fig. 1B). Results are in accordance with the recent observation that tissue-type is the most influent factor for variance in methylation datasets in ocular and blood tissues^12^. We plotted the correlation between retina samples from Hewitt and collaborators^12^ with our retina samples and also choroid/RPE samples with our choroid/RPE/ciliary body samples. In both cases, we described a high correlation under a Pearson test (p-val < 2e-16) (r^2^ = 0.992 and r^2^ = 0.993 for retina samples and choroid/RPE tissues, respectively) (Supplementary Fig. S1). Gene ontology (GO) analysis demonstrated that ret-CpGs specifically hypermethylated in the retina were mostly enriched for GO with respect to immune response, proliferation and angiogenesis (Fig. 1C), whereas hypomethylated CpGs in retina were mostly involved in regulating visual systems and photoreceptor function (Fig. 1C).

Retina is the tissue in which most ocular diseases throughout the world arise. For this reason, we decided to focus our studies on ret-CpGs. Considering the 40 retina-specific genes present in the Tissue Specific Genes Database, 24 contained a ret-CpG (odds ratio = 3.7, p < 0.001) (Supplementary Table S2). We selected four retina-specific genes that contain hypomethylated ret-CpG in their regulatory regions (defined as CpGs into first exon, 5′UTR or 1,5 KB from TSS): the calcium-binding protein that activates photoreceptor guanylate cyclases (GUCAB1); the photoreceptor-associated leucine-rich repeat transmembrane protein 1 (LRIT1); the rod-specific phosphodiesterase 6 A (PDE6A); and the potassium voltage-gated channel (KCNB1). Microarray data were validated by bisulfite sequencing of multiple clones at the GUCAB1, LRT1, PDE6 and KCNB1 regulatory regions (Supplementary Fig. S2). Importantly, hypermethylation of these four genes was significantly associated with a lower level of gene expression (Fig. 1D).

### Epigenetic regulation of transcription factors associated with retinogenesis in fetal and adult eyes

**Figure 2:**
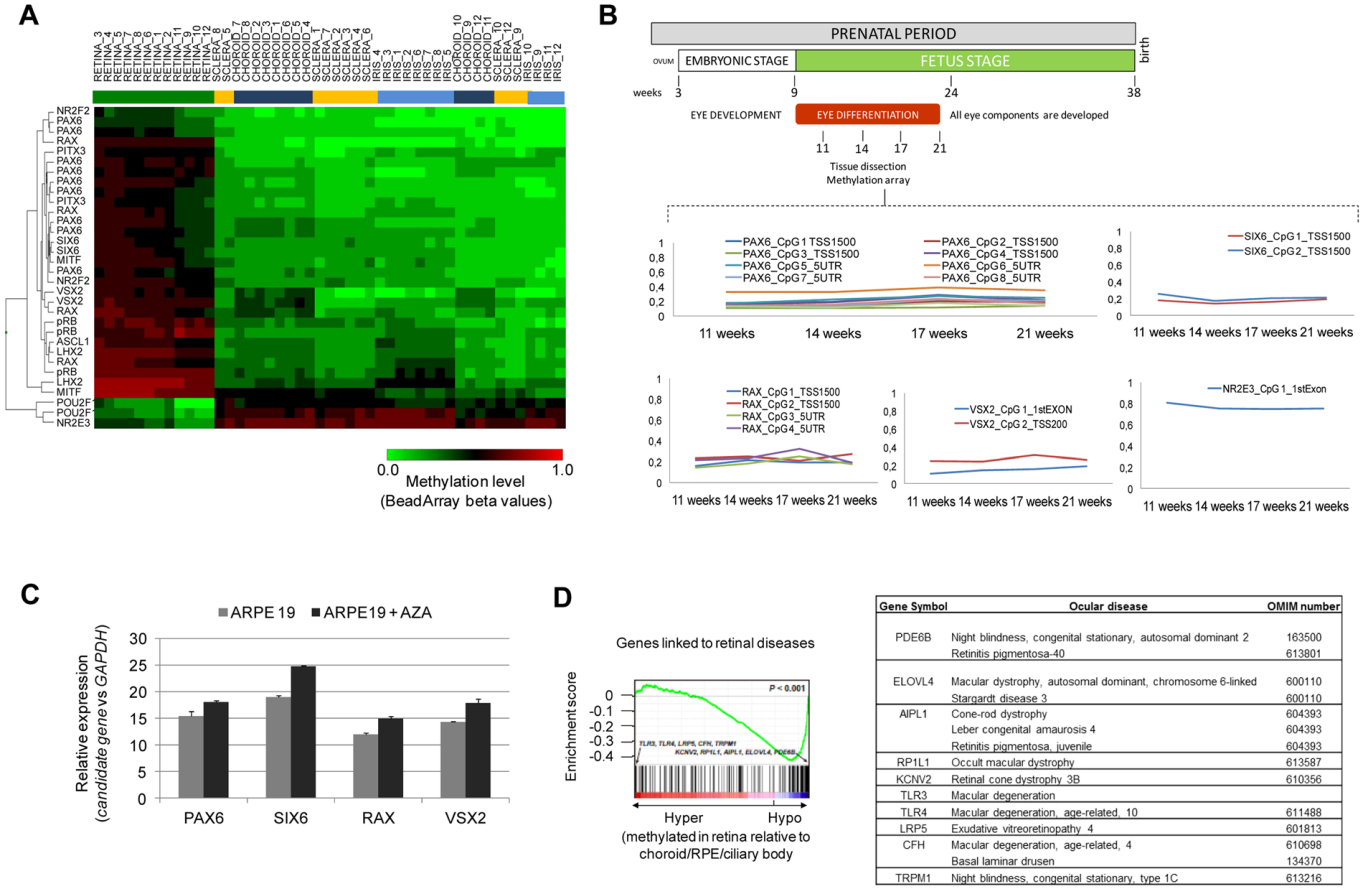
Epigenetic regulation of retinal transcription factors. (**A**) Supervised cluster heatmap using the most significant differences (methylation differences higher than 33% and a false-discovery rate < 0.01 in an ANOVA test-adjusted for multiple testing) among retina and the other tissues (sclera, iris and choroid/RPE/ciliary body) contained into transcription factors involved in retina development. (**B**) β-methylation values of PAX6, SIX6, RAX, VSX2 and NR2E3 retinal transcription factors obtained in retina from human fetus at weeks 11, 14, 17 and 21 of gestation. (**C**) Quantitative reverse transcription-PCR (qPCR) analysis of the expression of PAX6, SIX6, RAX and VSX2 transcription factors after and before treatment with the demethylating agent 5-aza-2′-deoxycytidine (AZA) in the hypermethylated retinal pigment epithelial cell line ARPE19. Average of three biological replicates and standard deviations are represented. (**D**) Gene set enrichment analysis (GSEA) of differentially methylated genes in retina samples with defined roles in ocular diseases. A summary of the most significant genes and their ocular diseases is shown.

In addition to epigenetic control, various transcription factors are known to execute essential activities in gene regulation in human retinal development^2,13^. We examined whether their expression could also be regulated by CpG methylation. We interrogated a representative list of 55 well-studied DNA-binding transcription factors that regulate retinal development and are represented into the Infinium 450 K methylation array^2^ and found that 42 transcription factors contained ret-CpGs in their regulatory regions (Supplementary Table S3). Supervised clustering of the ret-CpGs into TFs sequences showing more than a 33% of a methylation difference among retina and the other tissue-types allows the segregation into independent branches (Fig. 2A). Most of these transcription factors influence the fate of retinal cells during the early stages of their development; however, their level of expression is lower in adult retina (or is confined to specific cellular compartments such as neural stem cell niches)^14,15^. Accordingly, we found that the majority of these transcription factors were hypermethylated in adult retina, including the best-characterized transcription factors for retinal induction, such as paired box 6 (*PAX6*), the SIX homeobox family (*SIX3*, *SIX6)* and retinal homeobox transcription factors (RAX) (Fig. 2A, Supplementary Fig. S2). By contrast, only two of the transcription factors were hypomethylated in adult retina: the POU class 2 homeobox 1 (POU2F1) involved in lens development, and the nuclear receptor transcription factor NR2E3 that activates rhodopsin expression in adult retina (Fig. 2A). To complement these data, we obtained ocular globes for fetuses at different stages of gestation (11, 14, 17 and 21 weeks) and obtained the retina samples (Fig. 2B). It is important to note that human eyes develop at week 9 of gestation. At this point, the fetus possesses the layers of the adult eye although the visual structures mature during subsequent fetal development. We observed that transcription factors associated with retinal cell fate in progenitor cells (Six6, Vsx2, Rax6) and general neural induction (Pax6) were unmethylated during embryonic development, with no substantial changes during the four stages analyzed (Fig. 2B). In contrast, the NR2E3 gene with an active role in adult retina was methylated in fetus (Fig. 2B). To unravel the impact of promoter hypermethylation in the expression levels of PAX6, SIX6, RAX and VSX2 genes, we quantified their expression levels before and after treatment with the demethylating drug 5-aza-2′-deoxycytidine (AZA). After checking the PAX6, SIX6, RAX and VSX2 promoter hypermethylation in the retinal pigment epithelial cell line ARPE-19 (Fig. S2B) we quantified an increased gene expression after AZA treatment (Fig. 2C).

Finally, genetic mutations of more than 200 genes are known to be associated with retinal diseases (see RetNet database for additional details; https://sph.uth.edu/RetNet/home.html). Indeed, gene set enrichment analysis (GSEA) revealed a statistically significant correlation (p < 0.001) between the hypomethylated ret-CpGs and the genes associated with retinal diseases (Fig. 2D). Although the genetic basis of retinal disorders is being widely investigated, our knowledge of the epigenetic contribution to visual disorders is very limited. For this reason, we decided to explore the epigenetic changes associated with the most prevalent ocular disorders, including non-tumoral and tumoral ocular diseases.

### CpG methylation in diabetic retinopathy and proliferative vitreoretinopathy

**Figure 3:**
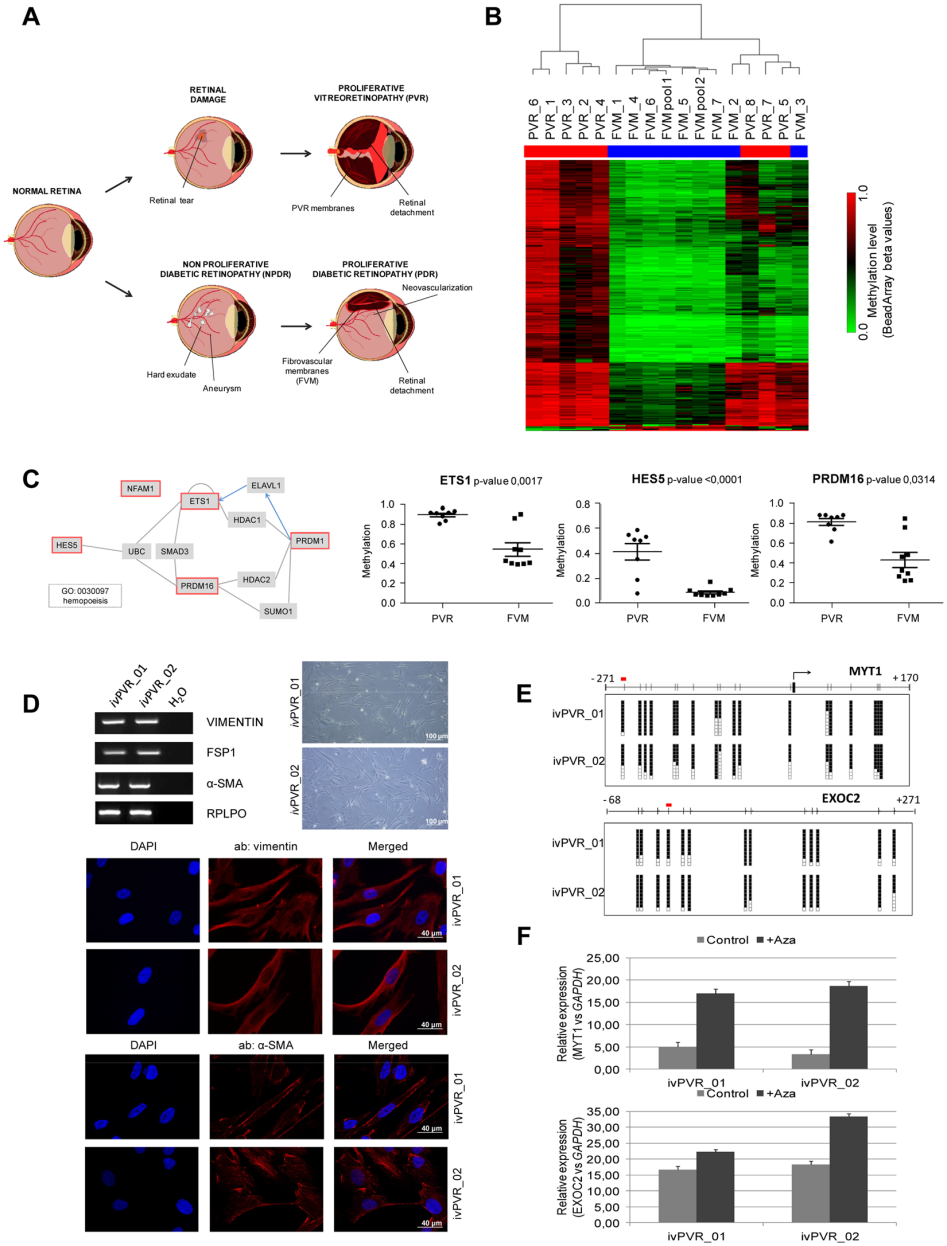
DNA methylation profile of fibrovascular membranes in diabetic retinopathy and rhegmatogenous retinal detachment. (**A**) Schematic depiction of the retinal detachment models used in the methylation study. *Above*, proliferative vitreoretinopathy (PVR) membranes were obtained from patients with rhegmatogenous retinal detachment due to traumatism or complications after cataract surgery. *Below*, the diabetic retinopathy model was composed of neuroretinas from non-proliferative diabetic retinopathy (NPDR) patients and fibrovascular membranes (FVM) in advanced proliferative disease. Normal neuroretinas were used as controls in both models. (**B**) Supervised hierarchical clustering using the 293 significantly differentially methylated CpGs between PVR and FVM membranes. (**C**) *Left*, interaction graph of genes from the hemopoiesis pathway containing FVM-CpGs in their regulatory regions. Blue arrows represent protein-RNA interactions; grey arrows indicate protein-protein interactions. Genes in red boxes are those containing FVM-CpGs. *Right*, significant methylation differences of ETS1, HES5 and PRDM16 genes between FVM and PVR membranes. (**D**) Characterization of *in vitro-*cultured fibroblasts from patients with PVR. Vitreous samples obtained from two patients at the outset of vitrectomy surgery were cultured under *in vitro* conditions. The resulting growing cells exhibit fibroblast morphology (*left*, *below*). Fibroblast markers (vimentin, fibroblast specific protein 1 (FSP1) and alpha-smooth muscle antigen (α-SMA) were studied by RT-PCR (*left*, *above*) and immunofluorescence (*right*). Ribosomal protein, large, P0 (RPLPO) genes were used as an endogenous control. (**E**) Bisulfite sequencing of MYT1 and EXOC2 promoter in *in vitro-*cultured fibroblasts from patients with PVR. CpG dinucleotides are represented as short vertical lines. Results of bisulfite genomic sequencing of 10 individual clones are shown. The presence of a methylated or unmethylated cytosine is indicated by a black or white square, respectively. The CpGs included in the methylation array are indicated by a red box. (**F**) Quantitative reverse transcription-PCR (qPCR) analysis of the expression of hypermethylated genes in PVR membranes (MYT1 and EXOC2) before and after treatment with the demethylating agent 5-aza-2′-deoxycytidine (AZA) in the hypermethylated *in vitro* PVR cell lines. Average of three biological replicates and standard deviations are represented. ivPVR; *in vitro* cells derived from proliferative vitreoretinopathy patients.

Diabetic retinopathy is a serious eye disease that occurs in people who have diabetes. The high sugar contents are associated with ischemic and exudative damage to retinal vessel causing visual loss and in latter stages of the disease, retinal detachment due to membrane formation and contraction. We developed a three-step model of diabetic retinopathy to study genome-wide CpG methylation during the progression of diabetic retinopathy in: (1) neuroretina from healthy donors (n = 8), (2) retina from background non-proliferative diabetic retinopathy (NPDR) with cotton-wool spots, microaneurysms but no retinal detachment (n = 8) and (3) fibrovascular membranes (FVMs) obtained from vitreoretinal surgery in patients with proliferative diabetic retinopathy (PDR) (n = 9) (Fig. 3A; Supplementary Fig. S3; Supplementary Table S1). Unsupervised clustering of normal neuroretinas and NPDR samples does not allow the segregation in two independent branches (Supplementary Fig. S3). After applying the statistical test, we observed that NPDR implies changes in CpG methylation of 46 genes with respect to normal retina (Supplementary Table S4). Interestingly, consistent with previous ref.^16^, we found significant enrichment in genes from the Wnt/β-catenin pathway (Supplementary Fig. S3) in the Top Canonical Pathways, according to our list of differentially methylation values in NPDR. As previous research has implicated p38 MAPK/NF-κB pathway in the pathogenesis of early stages of diabetic retinopathy^17^, we focused our attention on MAP3K1 methylation. We observed a tendency towards hypomethylation in MAP3K1 promoter in NPDR (Supplementary Fig. S3).

Advanced diabetic retinopathy involves the formation of fibrovascular membranes (FVMs) mainly composed of fibrotic tissue leading to retinal detachment. Another cause of retinal detachment is the formation of fibrotic membranes in proliferative vitreoretinopathy (PVR). This process can be a consequence of traumatisms or complications after retinal surgery (rhegmatogenous retinal detachment) (Fig. 3A). Inflammatory factors are involved in physiopathology of both diseases, but there are slight differences between FVM and PVR. As an example, cellular composition varies between PVR and FVM membranes: PVR membranes contain a greater proportion of proliferating cells with a high density of glial and immune cells than in FVM membranes^18^, whereas FVM membranes are characterized by neovascularization through the presence of vascular endothelial cells^19^. The selective alterations involved in early vascular changes in diabetic retinopathy patients (but not in PVR) could facilitate the design of novel therapeutic approaches for preventing FVM formation in proliferative diabetic retinopathy patients. For this reason, we decided to study the CpG methylation differences between FVM and PVR membranes. Only 293 CpGs were found to be differentially methylated between FVM and PVR membranes (Fig. 3B **;** Supplementary Dataset 1) and the main differences were associated with hypomethylation in FVM (287 out of 293). As expected, genes associated with hematopoietic processes were hypomethylated in FVM with respect to PVR, including transcription factors (ETS1, HES5) or proteins required for hematopoietic stem cell renewal and differentiation (PRDM16) (Fig. 3C) providing further evidence of the epigenetic control of angiogenesis in diabetic retinopathy progression.

Finally, we developed an *in vitro* PVR model to study the correlation between promoter hypermethylation and gene silencing. We obtained two vitreous samples at the outset of the vitrectomy surgery of patients with active PVR (Supplementary Table S1) that were established *in vitro*. As shown in Fig. 3D, most of the cell population after establishment corresponded to cells expressing biomarkers of active myofibroblast, such as fibroblast-specific protein 1 (FSP1) or α-smooth muscle actin (α-SMA). We selected genes highly methylated in PVR and FVM samples related to signal transduction and visual function: the myelin transcription factor 1 (MYT1) and the exocyst complex component 2 (EXOC2). After validating MYT1 and EXOC2 promoter hypermethylation in the *in vitro* system of PVR (*iv*PVR_01 and *iv*PVR_02) by bisulfite sequencing (Fig. 3E) we demonstrated that gene expression was reactivated after treatment with the demethylating drug 5-azacytidine (Fig. 3F).

### CpG methylation changes associated with uveal melanoma

**Figure 4:**
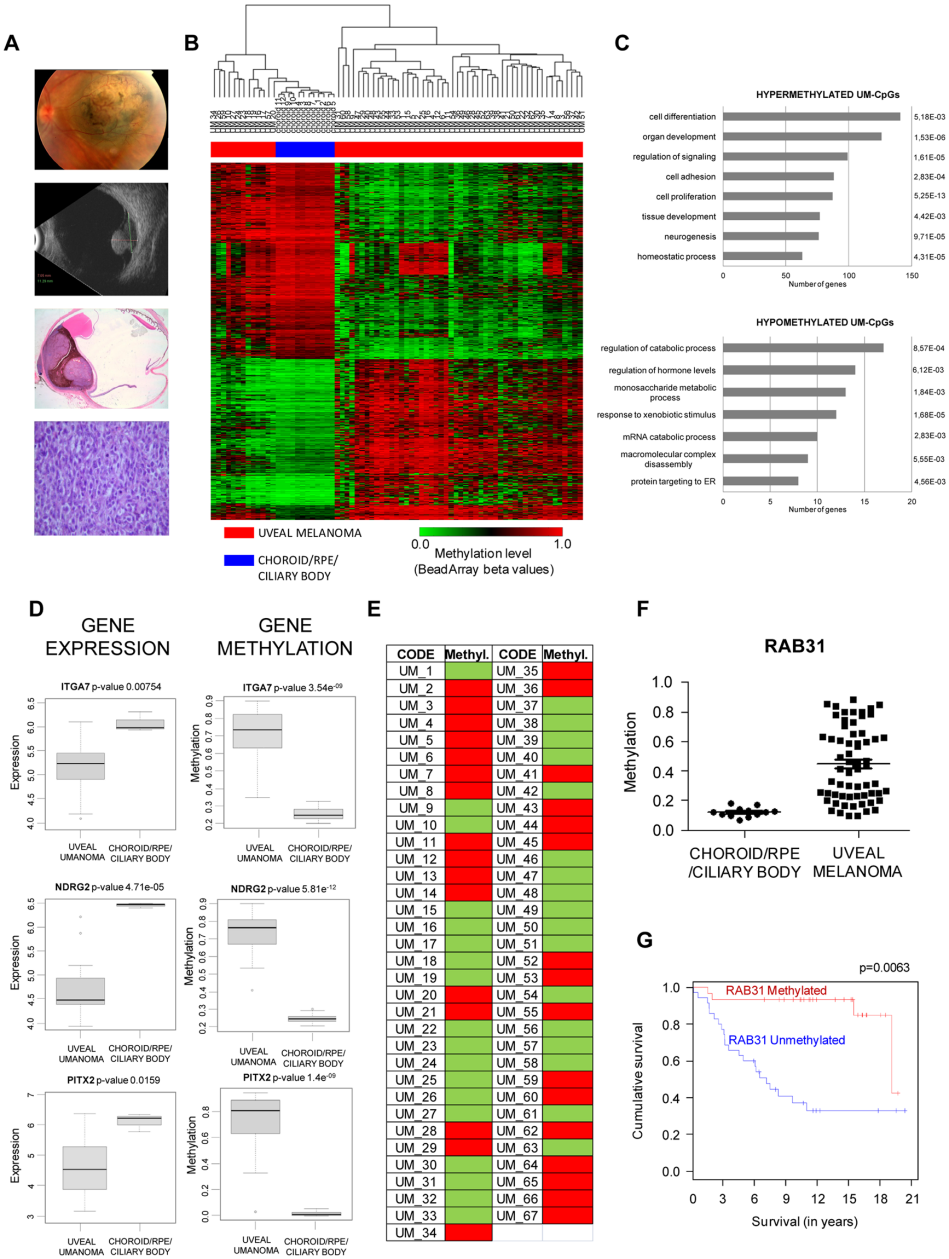
Epigenetic regulation in uveal melanoma. (**A**) *From up to down*, image of a choroidal melanoma in the posterior pole; an ultrasound image showing the Bruch membrane rupture secondary to tumor growth; example of an enucleated eye showing a mixed epithelioid spindle cell melanoma. (**B**) Supervised hierarchical clustering using the 1,841 significantly differentially methylated CpGs in uveal melanoma (UM-CpGs). (**C**) Gene ontology analysis of genes associated with hypermethylated UM-CpGs (*above*) and hypomethylated UM-CpGs (*below*). The length of each bar is proportional to the number of differentially expressed genes in the functional category. Numbers on the right represent the statistically significant p-values associated with a one-sided Fisher’s exact test. (**D**) Correlation analysis of gene expression data from GEO Omnibus database GSE51880 y GSE20986 (*left*) and CpG methylation value obtained from Infinium 450 K array (*right*) for *ITGA7*, *NDRG2* and *PITX2* genes. (**E**) Analysis of *RAB31* promoter methylation by methylation-specific PCR in a validation cohort of uveal melanoma samples (n = 67). Red and green squares indicate hypermethylated or unmethylated CpG region, respectively. (**F**) Methylation values for RAB31 in normal choroid/RPE/ciliary body and uveal melanoma inferred from methylation array. (**G**) Kaplan–Meier analysis of RAB31 promoter hypermethylation in uveal melanoma patients. RAB31 promoter hypermethylation was significantly associated with lower overall survival (p = 0.0063).

We examined the genome-wide methylome of human uveal melanoma (n = 63, Fig. 4A; Supplementary Table S1) and non-pathological choroid/RPE/ciliary body tissues (n = 12) as controls. As indicated in Supplementary Fig. S4 the unsupervised clustering of samples allows the segregation into two branches: tumors and controls. We identified 1,841 CpGs that were differentially methylated (termed UM-CpGs) (Fig. 4B, Supplementary Dataset 1). In contrast to the methylation changes associated with non-tumoral diseases, 45% of the UM-PVRs were hypermethylated in cancer (829 hypermethylated and 1012 hypomethylated CpGs in uveal melanoma). GO annotations of the hypermethylated UM-CpGs in cancer showed an enrichment of functions associated with cell differentiation, cell development and signal regulation (Fig. 4C). Indeed, considering the tumor suppressor gene TSG Database^20^, tumor suppressor genes in the list of hypermethylated UM-CpGs were significantly enriched (p = 0.043; Fisher’s exact test). We selected three genes on the basis of their roles in tumorigenesis: integrin alpha 7 subunit (*ITGA7*), *N-myc downstream-regulated gene 2* (*NDRG2)* gene and *paired-like homeodomain 2* (*PITX2*) for bisulfite sequencing validation (Supplementary Fig. S2). Notably, hypermethylation of *ITGA7*, *NDRG2* and *PITX2* was associated with decreased gene expression (Fig. 4D).

Next, we validated the methylation results in an independent cohort of uveal melanoma samples. We examined the methylation status of 67 human primary uveal melanoma tumors corresponding to different tumor stages (Supplementary Table S1) by methylation-specific PCR (MSP). Promoter hypermethylation was extensively observed in the ITGA7, NDRG2 and PITX2 genes (85% methylated samples) in uveal melanoma (Supplementary Fig. S4). The widespread hypermethylation of *ITGA7*, *NDRG2* and *PITX2* observed prompted us to consider whether it was correlated with clinicopathological and molecular features. We found no association between gene hypermethylation and age at diagnosis, gender, clinical stage, sclera invasion or cell type for these genes (Supplementary Fig. S4), suggesting that CpG hypermethylation of *ITGA7*, *NDRG2* and *PITX2* is an early phenomenon in uveal melanoma. A panel of biomarkers based on the gene expression profile of 12 genes has been proposed that could distinguish between low and high metastatic risk in uveal melanoma^21^. We evaluated the CpG methylation of the 12 genes included in the panel in our collection of primary uveal melanoma samples. Only one gene from this panel, RAB31, a member of the RAS oncogene family, showed differential methylation between normal uvea and uveal melanoma, although the methylation level in individual cancer patients was highly variable (Fig. 4F). We analyzed the methylation status in our retrospective cohort for which metastasis data were available (Fig. 4E). The Kaplan-Meier survival analysis of 67 primary uveal melanoma samples (Fig. 4G) demonstrated that RAB31 unmethylation is a predictor of poor outcome in uveal melanoma. In fact, the group of samples without RAB31 promoter methylation showed significantly lower overall survival (p = 0.0063) than did the hypermethylated samples.

### CpG methylation alterations in retinoblastoma

**Figure 5:**
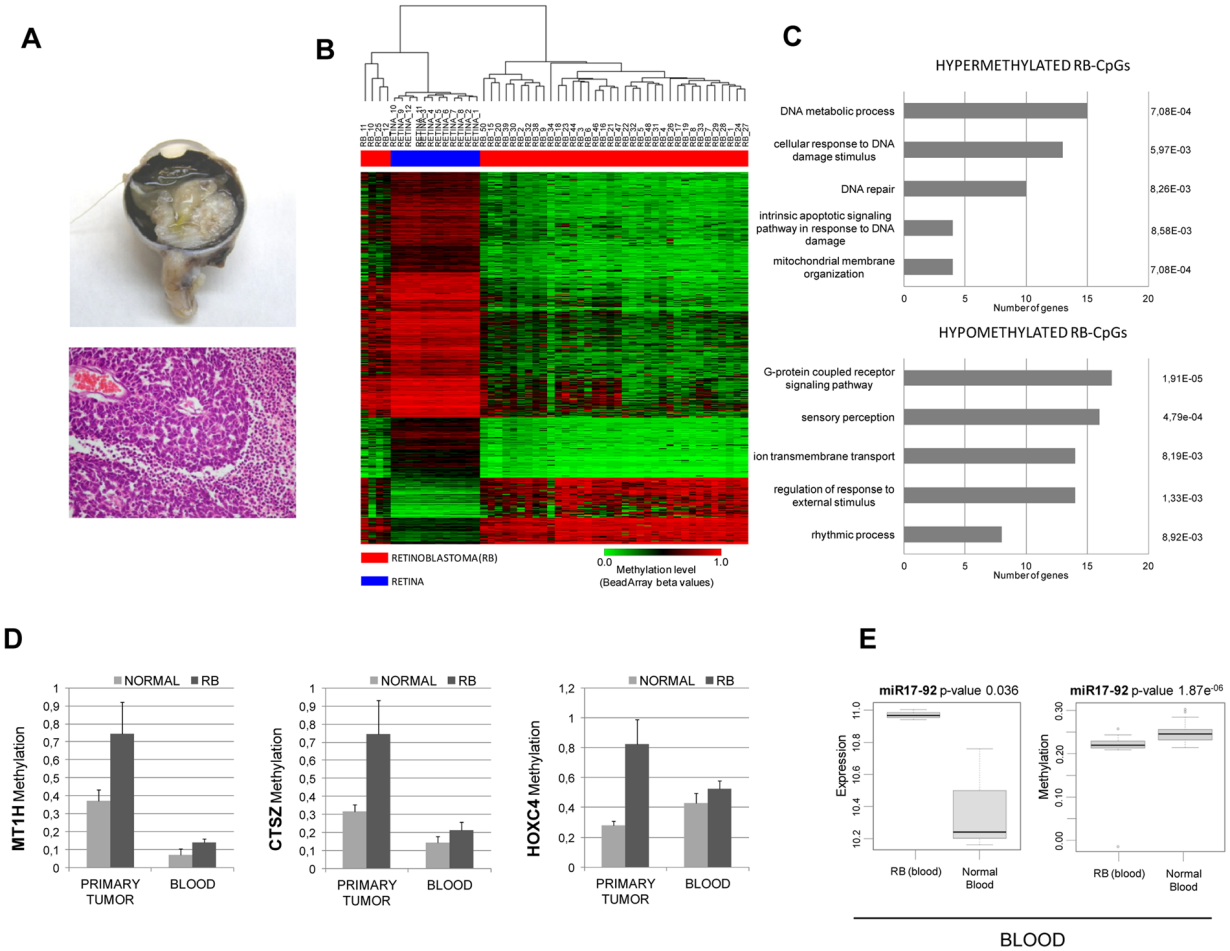
Retinoblastoma has a specific CpG methylation signature in primary tumors and blood. (**A**) Representative retinoblastoma from stage E occupying more than half of the eye that was finally enucleated. Pathological studies disclosed a moderately differentiated retinoblastoma with areas of necrosis (hematoxylin and eosin staining). (**B**) Supervised hierarchical clustering approach using the 15,428 significantly differentially methylated CpGs in primary tumors from retinoblastoma (RB-CpGs). (**C**) Gene ontology analysis of genes associated with hypermethylated RB-CpGs (*above*) and hypomethylated RB-CpGs (*below*). The length of each bar is proportional to the number of differentially expressed genes in the functional category. Numbers on the right represent the statistically significant p-values associated with a one-sided Fisher’s exact test. (**D**) Three representative genes (MT1H, CTSZ and HOXC4) with increased methylation levels in retinoblastoma samples (from tumor and blood) with respect to their normal counterparts. (**E**) Gene expression (*left*) and ß-methylation (*right*) values obtained from the oncogenic miR17-92 cluster in blood from retinoblastoma patients and healthy children.

As a second model of ocular cancer, we analyzed a large cohort of retinoblastoma samples (n = 40) and their normal retina controls (n = 12) (Fig. 5A; Supplementary Table S1). Furthermore, we assessed whether this epigenetic deregulation could also be detected in peripheral blood samples from retinoblastoma patients (n = 27) (Supplementary Table S1). Aging is an influent factor on the variance in the CpG methylation in our dataset of retinoblastoma patients (pediatric) and controls (adult retina) (Supplementary Fig. S6). In consequence, we correct the methylation values based on aging and identified 554 CpGs that change their value between retinoblastoma and normal retina (termed RB-CpGs) (Supplementary Dataset 1). As the retina is a high methylated tissue, most of the CpG methylation changes were losses of methylation (169 hypermethylated and 385 hypomethylated CpGs in retinoblastoma). GO annotations of the hypermethylated RB-CpGs in cancer showed an enrichment of the functions associated with DNA metabolic processes, DNA repair or response to DNA damage stimulus (Fig. 5C). On the other hand, hypomethylated genes were associated with protein signaling transduction, sensory perception or response to external stimulus (Fig. 5C).

Epigenetic biomarkers detected in non-invasive samples are especially needed in pediatric oncology. Taking this into consideration, we set out to identify how many of the CpG methylation detected in primary tumors could also be detected in blood samples from retinoblastoma patients. As CpG methylation has a tissue-specific pattern, we used methylation data obtained from blood of healthy children aged 3 to 17 years^22^ (GSE 36064) as controls. As expected, genome-wide profiles of retinoblastoma samples from blood and primary tumors could be completely segregated by principal components analysis (Supplementary Fig. S5). Furthermore, the retinoblastoma methylation signature observed in blood revealed small methylation differences (up to 30%) between patients and healthy donors (Supplementary Dataset 1). The distribution of the differences between normal and retinoblastoma methylation values in blood also reflects a loss of methylation in the blood from retinoblastoma patients with respect to control samples (Supplementary Fig. S5). Interestingly, 939 CpGs were present in both the list of RB-CpGs in primary tumors and the retinoblastoma-specific CpGs detected in the blood samples (Supplementary Dataset 1). Figure 5D shows the methylation values of the three most significantly hypermethylated genes both in primary tumors and blood samples from retinoblastoma patients: metallothionein 1 H (MT1H), cathepsin Z (CTSZ) and homeobox C4 (HOXC4). CpG methylation in miRNAs was also detected (Supplementary Dataset 1). Due to its oncogenic role, we decided to study the methylation levels of miR-17-92. We observed that miR-17-92 cluster has a loss of methylation associated with the greater miRNA expression in blood from retinoblastoma patients (Fig. 5E). Common CpG methylation changes in tumors and blood from patients with retinoblastoma relative to healthy controls reinforced the idea that the retinoblastoma has a specific methylation signature whose translational uses need to be explored.

## Discussion

**Figure 6:**
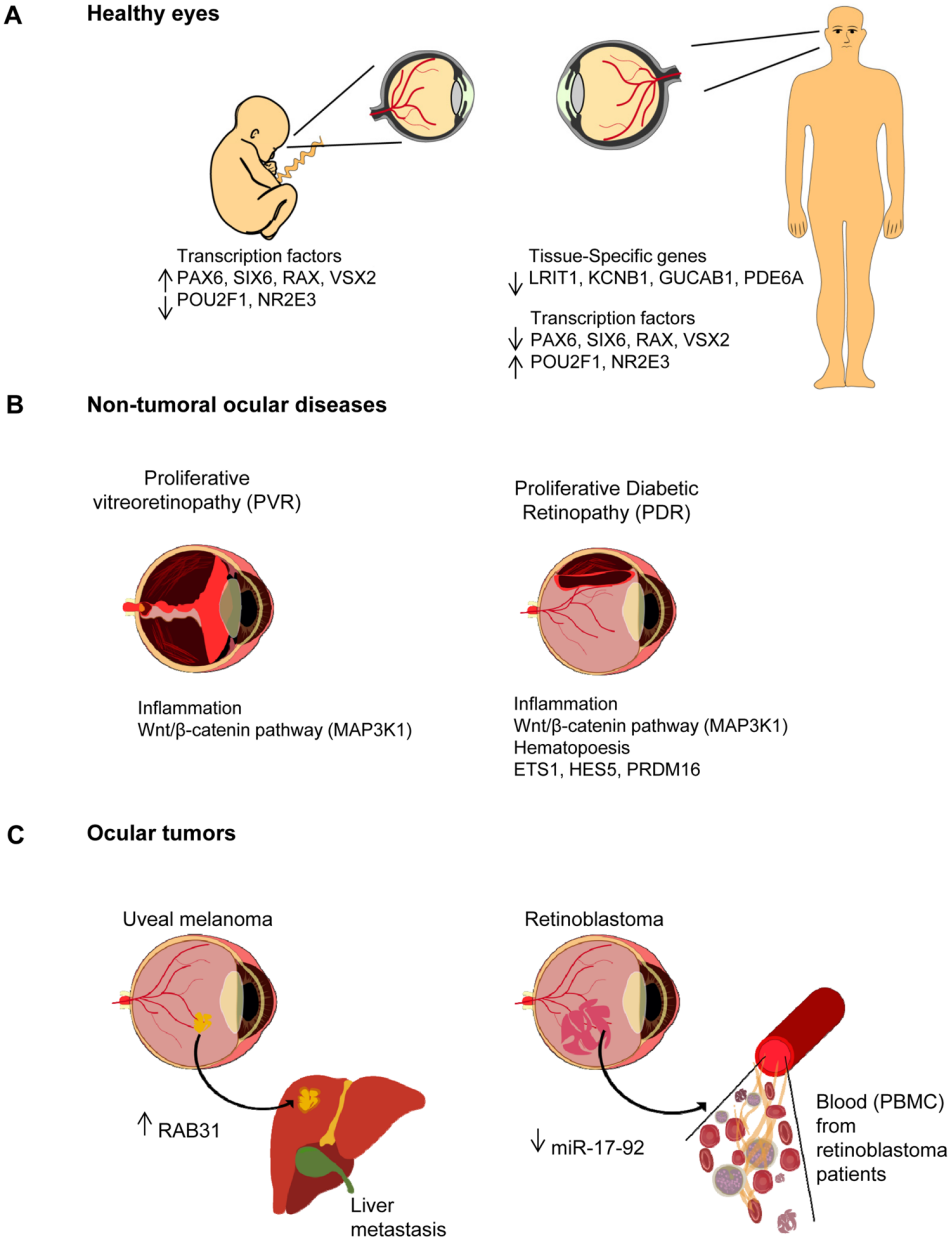
Genome-wide CpG methylation screening reveals pathways controlling tissue specificity and ocular-associated disorders. Schematic representation of our proposed candidate genes regulated by CpG methylation that may play a crucial role in maintaining tissue specificity in the eye and in ocular disorders. (**A**) CpG methylation-mediated silencing of retinal transcription factors from the PAX6 network was found in adult retina. (**B**) *Left panel*, inflammation-related genes are epigenetically regulated in fibrotic membranes causing rhegmatogenous retinal detachment (PVR). *Right panel*, in addition to inflammation- associated genes, CpG methylation of genes involved in the formation of new blood vessels reinforce the role of neovascularization in diabetic retinopathy and its therapeutic use; (**C**) *Left panel*, loss of methylation of the RAB31 gene is associated with poor survival and metastatic uveal melanoma; *right panel*, up-expression of the oncogenic miR-17-92 cluster is linked to loss of methylation in retinoblastoma patients. This epigenetic alteration was also detected in blood samples from retinoblastoma patients.

The retina is an immensely complex structure in which the orchestrated control of gene expression by epigenetic regulation is of great importance. It has been previously described that global reductions of epigenetic enzymes impair normal development of retinal cells^5–7^ and that CpG methylation is a mechanism for controlling retinal specification^1,12^. In this study we have described specific methylation at CpGs located in visual associated genes (e.g., GUCA1B, LRIT1, KCNB1 and PDE6A) in retina samples that differ from the methylation level in the other ocular tissues (Fig. 6A). The most “prominent” eye gene is probably the transcription factor PAX6^23,24^. PAX6 may act as both a gene activator and a repressor and its expression level varies depending on the tissue and the state of development^23,25^. We found that CpG hypermethylation mediates silencing of PAX6 and its associated TFs in mature retina but not in the other eye tissues. Furthermore, this hypermethylation was not found early in development (fetuses at 11 to 21 weeks of gestation) (Fig. 6A). The spatial and temporal control of TFs expression is a requisite for retina specification. In accordance with this observation, silencing of Six6 or Rax occurs after the establishment of the eye field and prevents formation of ectopic eye structures^26^. To address the role of CpG methylation in retinal specification several questions need to be investigated. On one hand, it is yet to be determined when the epigenetic changes occur, whether later on in fetal development or after birth, when complete visual function appears (postnatal weeks 5 to 7). In addition, functional assays should be performed to demonstrate the causal role of epigenetics in instructing gene expression in retinal tissues.

Diabetic retinopathy is one of the best models for studying the role of epigenetics as a bridge between metabolic diseases and environment (diet). It has been previously described that low levels of serum folic acid and vitamin B12 (intermediates for DNA methylation that are found in many common nutrients) increase the risk of diabetic retinopathy^27^ and that global DNA methylation levels are associated with retinopathy in diabetic patients with progression in accordance with the severity of the disease^28^. In this study, we found that CpG methylation regulates pathways that are used as pharmacological targets in diabetic retinopathy progression, such as angiogenesis (ETS1, HES5, PRDM16) (Fig. 6B). Although anti-VEGF therapies are beneficial in some cases of proliferative diabetic retinopathy^29^, the short duration of the effect has been reported^28^. Our results suggest new avenues of research into the potential synergic effect of epigenetic-based treatments in conjunction with conventional anti-angiogenesis drugs.

In general, the prognosis of uveal melanoma patients once the tumor has spread from the eye to the liver is very poor; no effective treatment for advanced uveal melanoma exists at present. Novel targets for clinical trials are emerging to increase therapy options for uveal melanoma patients with metastasis^30^. Epigenetic knowledge is also making its contribution and a clinical trial with the histone-deacetylase inhibitor vorinostat (NCT01587352) is in progress. In addition, development of appropriate clinical trials requires the use of biomarkers that predict drug response. On the basis of their gene expression profile, uveal melanomas have been classified into those at low risk of metastasis (class 1) and those at high risk (class 2)^21^. We found that the promoter methylation of one of the genes (RAB31) included in the panel of genes associated with metastasis was also correlated with patient survival (Fig. 6C). Specifically, unmethylation of the RAB31 promoter is a predictor of poor outcome in uveal melanoma. Together with gene expression profiles, this finding may be clinically relevant in the management of these patients as a prognostic factor. Furthermore, a DNA-based marker may be preferable to RNA-based methods because such samples are easier to maintain for analytical purposes.

A study of retinoblastoma demonstrated for the first time that epigenetic alterations may be a major driver of the disease, rather than the classic genetic lesions^31^.The findings of our study, which included the largest collection of retinoblastoma primary samples so far examined, are in accordance with main observations of Zhang and collaborators. In addition, we demonstrate that CpG methylation changes are also found in the blood of retinoblastoma patients. As the most notable example, we identify epigenetic deregulation of the mir-17-92 cluster in retinoblastoma in blood and primary tumors from patients (Fig. 6C). miR-17-92, also known as oncomir-1, is among the most potent oncogenic miRNAs^32^. Its increased expression is associated with aggressiveness and proliferation of several tumor types, including retinoblastoma^33^. Genetic inactivation of the miR-17-92 cluster is sufficient to prevent retinoblastoma formation in *in vivo* murine models, similarly to the effect of Rb, p53 or Dicer inactivation^34^.

In summary, our work, and its associated freely accessible GEO database, provides a useful CpG methylation atlas for the section of the scientific community who are interested in gene regulation in the field of ophthalmology. The approach used in this study enriches for CpGs located in coding regions, however there are probably more methylation differences between CpGs found at intergenic regions, such as enhancers, that were missed and deserve further exploration. Through the discovery of new molecular pathways that are altered in ocular diseases our results give rise to a challenging scenario for clinical and basic research in eye diseases that lies beyond the “classic” pathways.

## Methods

### Subject collection

The study has been approved by the Ethics Committee at Bellvitge University Hospital (Ref. PR181/12 and PR132/14). All donors gave informed consent before being included in the study. Surgical specimens were handled in accordance with the tenets of the Declaration of Helsinki.

Human whole eyes were obtained from donors of the Banc de Sang i Teixits (BST) of the Catalan Ministry of Health (Barcelona, Spain). The cause of death in all adult subjects was multiorgan dysfunction and none of them had exhibited previous ocular diseases (see Supplementary Table S1). Whole eyes were preserved in ice until further dissection into specific tissues. Average postmortem time was 4.6 hours. Manual dissection of the eyes allows the isolation of the following tissue types: (i) iris, (ii) choroid/RPE/ciliary body, (iii) sclera and (iv) retina. Fetal tissue at 11, 14, 17 and 21 weeks of gestation were obtained from the Biobank of the Hospital Universitario Vall d’Hebron. Dissected tissues were immediately frozen in liquid nitrogen and maintained at −80 °C until required. DNA extraction was performed individually for each eye and tissue.

Proliferative vitreoretinopathy (PVR) membranes were obtained from patients undergoing PVR surgery at the Retina Unit of the Bellvitge University Hospital (Barcelona, Spain). PVR membranes were surgically dissected from the retinal surface with Eckardt horizontal forceps in one eye of each of eight patients with a PVR diagnosis (see Supplementary Table S1 for clinical summary).

Diabetic retinopathy samples were obtained from patients at the Hospital Universitario Vall d’Hebron (Barcelona, Spain). Eight human postmortem eyes were obtained from diabetic donors free of fundoscopic abnormalities or with mild non-proliferative diabetic retinopathy in the ophthalmological examinations performed during the preceding two years. Eight eye-cups obtained from non-diabetic donors matched by age were used as a control group. The clinical characteristics of diabetic and non-diabetic donors included in the study are shown in Supplementary Table S1. The time elapsed from death to eye enucleation was 3.9 ± 1.5 h. After enucleation, one eye from each donor was snap-frozen in liquid nitrogen and stored at −80° until used. The other eye-cup was fixed in 4% paraformaldehyde and embedded in paraffin for the immunohistochemical study.

The fibrovascular membranes (FVM) obtained from diabetic patients FVMs were surgically dissected from the retinal surface in 12 eyes from 12 patients with type 2 diabetes and proliferative diabetic retinopathy at the Kyushu University Hospital (Japan), Fukuoka University Chikushi Hospital (Japan) and Bellvitge University Hospital (Barcelona, Spain) (see Supplementary Table S1 for clinical details). Due to limitations with the material, we obtained enough DNA to analyze seven samples individually, while five samples had to be pooled in two groups.

Uveal melanoma samples (n = 63) were obtained from patients at Bellvitge University Hospital (Barcelona, Spain) and the Shiley Eye Institute and Institute for Genomic Medicine (San Diego, USA). Fresh samples were immediately frozen after surgery and a fraction was conserved in the Anatomical Pathology Unit for histopathological characterization. Clinical features of the tumors are summarized in Supplementary Table S1. Paraffin-embedded samples corresponding to uveal melanoma tumors (n = 67) were also obtained from a collection of the Anatomical Pathology Unit at Bellvitge University Hospital (Barcelona, Spain) (see Supplementary Table S1).

Frozen retinoblastoma samples (n = 40) were obtained from patients at Sant Joan de Deu Hospital (HSJD) (n = 12, Barcelona, Spain), Instituto de Salud Carlos III (n = 15, Majadahonda; Madrid), Vall d’Hebron Hospital (HUVH) (n = 1; Barcelona, Spain), the Tumour Bank of the Spanish National Cancer Research Centre (n = 2) and the Pediatric Ophthalmology Service of the Pediatric Hospital EHS of Canastelin (Oran, Algeria) (n = 10). A collection of 27 blood samples from retinoblastoma obtained at the Pediatric Ophthalmology Service at the Pediatric Hospital EHS of Canastelin were also included in the study. Clinical features of the samples included in the study are summarized in Supplementary Table S1.

### *In vitro* establishment of fibroblasts from proliferative vitreoretinopathy

All human vitreous specimens were obtained from patients of the Retina Unit at the Bellvitge University Hospital (Barcelona, Spain). Briefly, vitreous samples were obtained at the outset of vitrectomy surgery of two patients. 0.2 mL of vitreous fluid was drawn from the central portion of the vitreous cavity after dry vitrectomy and collected using a TB syringe connected to the aspiration line. After centrifugation of the vitreous sample, the cellular pellet was treated with ammonium-chloride-potassium (ACK) lysis buffer (0.15 M NH_4_Cl; 1 mM KHCO_3_; 0.1 mM Na_2_EDTA) for abolishing blood cells. The resulting cells were cultured in DMEM Ham’s F12 medium (Invitrogen-Gibco) supplemented with 20% FBS 500 U/mL of penicillin, and 500 μg/mL of streptomycin. Cells were cultured at 37 °C in a humidified 5% CO_2_ atmosphere. After 15 days, cultures were washed with PBS buffer to remove unattached cells and then established fibroblasts were fed with fresh culture medium. Fibroblast markers were studied by RT-PCR for vimentin, fibroblast-specific protein 1 (FSP1), alpha-smooth muscle antigen (α-SMA) and ribosomal protein, large, P0 (RPLPO) genes, and by immunofluorescence, for vimentin and α-SMA. Primer sequences are listed in Supplementary Table S5.

### Cell line culture and DNA demethylation treatments

The ARPE-19 human retinal pigmented epithelium cell line examined in this study was obtained from the American Type Culture Collection (ATCC) (Rockland, MD, USA). Cell lines were maintained in appropriate media and treated with 1 μM 5-aza-2′-deoxycytidine (Sigma) for 72 h to achieve demethylation.

### DNA extraction, bisulfite modification and Infinium 450 K Methylation Array

DNAs were extracted using conventional phenol:chloroform:isoamylalcohol (Sigma), quantified by Quant-iT PicoGreen dsDNA Reagent (Invitrogen) and DNA integrity was analyzed in a 1.3% agarose gel. Bisulfite modification of 600 ng genomic DNA was carried out with the EZ DNA Methylation Kit (Zymo) following the manufacturer’s protocol. Next, 4 μL of bisulfite-converted DNA were used to hybridize on an Infinium HumanMethylation450 BeadChip, following the Illumina Infinium HD Methylation protocol. The chip was analyzed using Illumina HiScan SQ fluorescent scanner and the intensities of the images were extracted using GenomeStudio (2010.3) Methylation module (1.8.5) software. A three-step-based normalization procedure was performed using the lumi^35^ package available for Bioconductor^36^ in the R statistical environment^37^. This consisted of color bias adjustment, background level adjustment and quantile normalization across arrays^35^. The methylation level (β) for each of the 485,577 CpG sites was calculated as the ratio of methylated signal divided by the sum of methylated and unmethylated signals plus 100. To avoid batch effects, the function preprocessFunnorm in minfi package was used^38^. It is particularly useful for studies comparing conditions with known large-scale differences, such as cancer/normal studies, or between-tissue studies. It has been shown that for such studies, functional normalization outperforms other existing approaches^39^. After the normalization step, probes related to X and Y chromosomes, and those containing SNPs with a frequency of >1% (1000 Genome Project) in the probe sequence or interrogated CpG site were removed. Probes located in frequent copy number variant regions were also excluded. The methylation score of each CpG is represented as a β-value. DNA methylation microarray data are freely available for download from NCBI Gene Expression Omnibus under accession number GSE57362 (private link for the reviewers until the acceptance of the manuscript is https://www.ncbi.nlm.nih.gov/geo/query/acc.cgi?token = qdcvqcsyjbwpbid&acc = GSE57362).

### Hierarchical cluster analysis and definition of CpG methylation differences

Samples were clustered in an unsupervised manner using the 5,000 most variable β-values for CpG methylation according to their standard deviation in the CpG sites located in promoter regions by hierarchical clustering. An agglomeration method for Manhattan distances was used. Principal component analysis (PCA) was performed to analyze the effect of sample traits (age and tissue type) effect on the CpG methylation values among all sample set). Principal components analyses were run using the princomp package in the open-source R statistical software. This has been done for various sample traits such as age and tissue type. Also, projections of ages on the first two principal components (PC1 and PC2) were used for visualization of possible effects of age in distribution of the samples. For the differential methylation analysis between conditions (see main text), Wilcoxon signed-rank tests were conducted in the R statistical environment for all CpGs. The resulting p-values were corrected for multiple testing^40^. The CpGs selected were those with adjusted values of p < 0.05 and an absolute methylation differential value of >0.33.

### Gene ontology analysis

We employed the Bioconductor package GOStats^41^ in the R statistical environment to search for overrepresented gene ontology biological processes using Fisher’s exact test to obtaining probabilities for each pathway, thereby to control the error rate. We corrected for multiple testing with the Benjamini and Hochberg algorithm.

### Statistical analysis of correlations between CpG methylation and gene expression data

The following expression data from the GEO Omnibus database were downloaded in order to extract the normalized expression values from the differentially methylated samples in each condition: GSE29801 (for normal retina and choroid), GSE51880 and GSE20986 (for uveal melanoma) and GSE5222 (for retinoblastoma). All data were downloaded and analyzed in the R environment using the Bioconductor package GEOquery^42^. When a specific comparison demanded two expression arrays from different platforms because the data were not present in the GSE to enable comparison of the two tissues, we used the Bioconductor Package virtualArray^43^, which enables the combination of raw expression data of different microarray platforms regardless of the platform and chip generation method used. The package generates a combined virtual array as an”expression set” object from different datasets by matching raw data entries based on probe, transcript, gene and protein identifiers. Redundancies, gaps, and batch effects are removed before proceeding with data analysis.

### Uveal melanoma miRNASeq data

MirSeq data were downloaded from GEO Omnibus database under GSE18381 and GSE7072 for uveal melanoma and retinoblastoma samples, respectively. Fastq raw data were aligned to mature miRs from mirbase^44^ using NOVOALIGN software (http://www.novocraft.com/main/downloadpage.php). Raw counts for each miRNA were extracted and analyzed under using the Rsamtools and DESeq Bioconductor packages in R.

### Bisulfite genomic sequencing of multiple clones and methylation-specific PCR

We determined the CpG island methylation status of the selected genes by PCR analysis of bisulfite-modified genomic DNA, which induces the chemical conversion of unmethylated, but not methylated, cytosine to uracil. For bisulfite sequencing, we used the same cohort of samples than those used in the genome-wide analysis by methylation arrays. The candidate CpG contained in the methylation array was included into the PCR amplicon. After PCR and cloning, ten clones of each sequence and sample were automatically sequenced to determine their degree of methylation. Primer sequences are listed in Supplementary Table S5.

As a second strategy for assessing the CpG methylation status of specific genes in a panel of primary tumors from melanoma samples (n = 67) we used methylation-specific PCR using primers specific to either the methylated or modified unmethylated DNA. DNA from normal lymphocytes *in vitro* treated with *SssI* methyltransferase was used as a positive control for methylated alleles. DNA from normal lymphocytes was used as a positive control for unmethylated alleles. Primer sequences are listed in Supplementary Table S5.

### Quantitative RT-PCR (qPCR) expression analysis

Total RNA was prepared from all samples using TRIZOL® (Invitrogen, Carlsbad, CA) and further purified using RNeasy columns (Qiagen, GmbH) according to the manufacturer’s instructions. For qPCR assays, we reverse-transcribed total RNA (2 µg) treated with DNase I (Ambion) using oligo (dT) 20 primer with ThermoScript TM RT-PCR (Invitrogen). We carried out PCR reactions in a final volume of 16 µL containing 10x PCR buffer (Ecogen), 50 mM of MgCl_2_, 2 mM of dNTP, 1 µM of each primer and 3U of EcoStart DNA polymerase (Ecogen). 100 ng of cDNA were used for each PCR amplification. We also carried out PCR with GAPDH (22 cycles) to ensure cDNA quality and loading accuracy. Three biological replicates were included for each gene. Primer sequences are listed in Supplementary Table S5.

### Immunofluorescence

For immunolocalization experiments, cells were grown on coverslips and, after treatment, fixed with freshly prepared 4% paraformaldehyde for 20 min at RT or with 100% cold methanol for 10 min at 20°CC. Cells were mildly permeabilized with PBS containing 0.1% Triton and 2% bovine serum albumin at room temperature for 1 h. Thereafter, incubation with primary antibodies diluted in PBS with 0.1% bovine serum albumin was performed overnight, washed intensively and incubated with adequate secondary antibodies labeled with Alexa 488 and Alexa 568 dyes (Molecular Probes, Invitrogen). After staining, coverslips were mounted in Mowiol (Calbiochem, Darmstadt, Germany) with DAPI (Sigma). Images were acquired using a confocal spectral Leica SP5 microscope (Leica, Milton Keynes, UK). Z-projection and image processing were performed using ImageJ software (Macbiophotonics, Hamilton, ON, Canada). Primary antibodies and dilutions were: vimentin (Abcam) at 1:200 dilution and alpha smooth muscle actin (Abcam) at 1:200 dilution.

### Terminal transferase dUTP nick-end labeling (TUNEL)

Paraffin-embedded eye sections (7-μm thick) from diabetic and non-diabetic donors were processed with an apoptosis detection kit (APO-BrdU TUNEL Assay kit; Molecular Probes; Eugene, OR, USA). TUNEL staining in the diabetic retina was compared with that in the non-diabetic retina. For this purpose, each retina was visually scanned with a high power lens (60x), which covers an area of 212 × 212 μm.

### Availability of Data And Materials

The DNA methylation microarray data reported in this article can be found at Gene Expression Ommibus (GEO) under the accession number GSE57362. (private link for the reviewers until the acceptance of the manuscript is https://www.ncbi.nlm.nih.gov/geo/query/acc.cgi?token = qdcvqcsyjbwpbid&acc = GSE57362).

## Electronic supplementary material

Supplementary Information

Supplementary Dataset 1
