# Supplementary material for: DNA Methylomes Reveal Biological Networks Involved in Human Eye Development, Functions and Associated Disorders
Source: Sci Rep. 2017 Sep 18;7:11762. doi: 10.1038/s41598-017-12084-1 (PMC5603607; doi:10.1038/s41598-017-12084-1)
Supplement: Supplementary file 1 — Supplementary Information [file 41598_2017_12084_MOESM1_ESM.pdf]

INVENTORY OF SUPPLEMENTARY INFORMATION

DNA Methylomes Reveal Biological Networks Involved in Human Eye Development, Functions and Associated Disorders

María Berdasco[1,*], Antonio Gómez[1], Marcos J. Rubio[2], Jaume Català-Mora[3], Vicente Zanón-Moreno[4], Miguel Lopez[1], Cristina Hernández[6], Shigeo Yoshida[7], Takahito Nakama[7], Keijiro Ishikawa[7], Tatsuro Ishibashi[7], Amina M. Boubekeur[8], Lotfi Louhibi[8], Miguel A Pujana[9], Sergi Sayols[1], Fernando Setien[1], Dolores Corella[4], Carmen de Torres[3], Andreu Parareda[3], Jaume Mora[3], Ling Zhao[10], Kang Zhang[10], Matilde E. Lleonart[11], Javier Alonso[5], Rafael Simó[6,12], Josep M. Caminal[2] and Manel Esteller[1,13,14*]

This file contains:

- Supplementary Fig.1
- Supplementary Fig. 2
- Supplementary Fig. 3
- Supplementary Fig. 4
- Supplementary Fig.5
- Supplementary Fig.6
- Supplementary Table S1
- Supplementary Table S2
- Supplementary Table S3
- Supplementary Table S4
- Supplementary Table S5

Supplementary Fig. S1

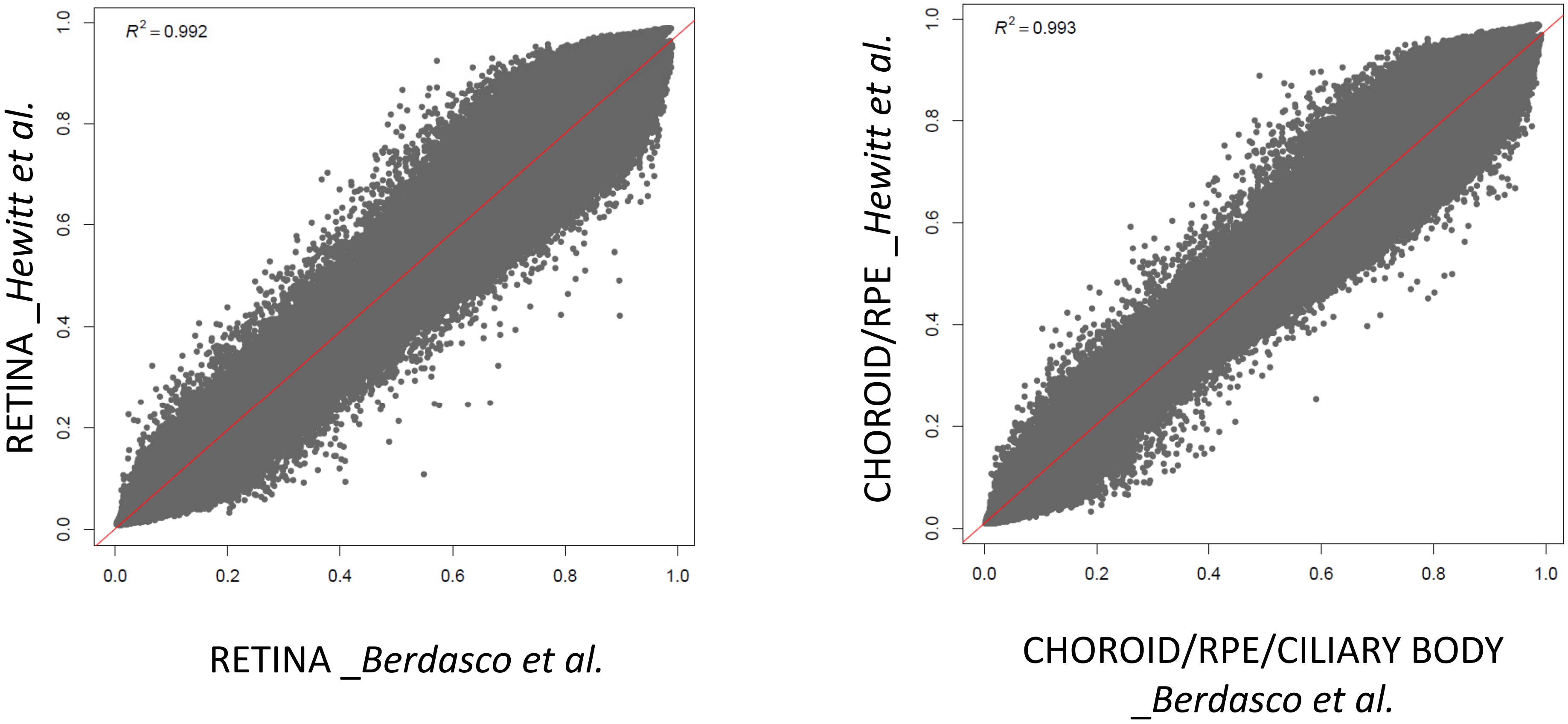

Supplementary Fig. S2

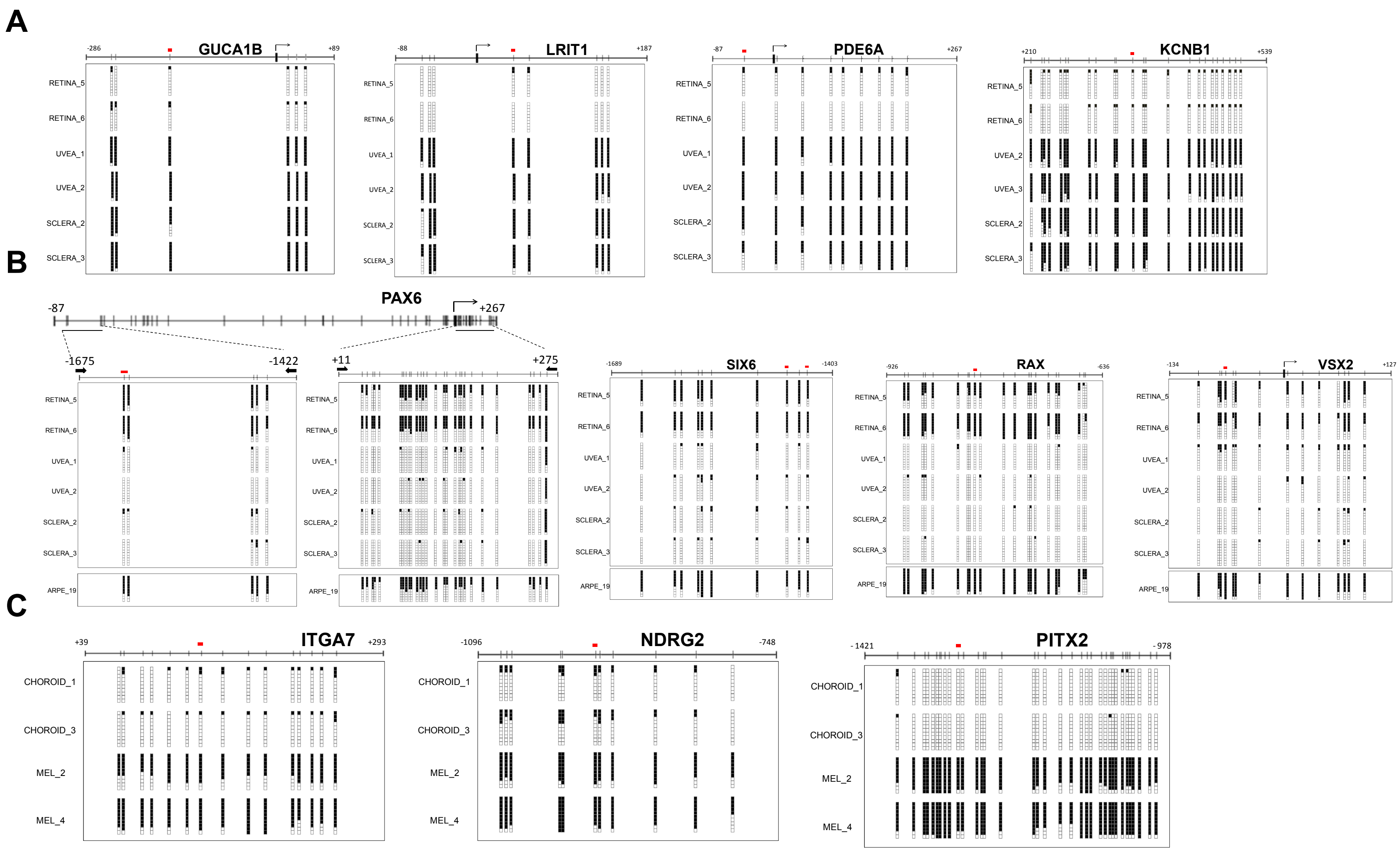

Supplementary Fig. S3

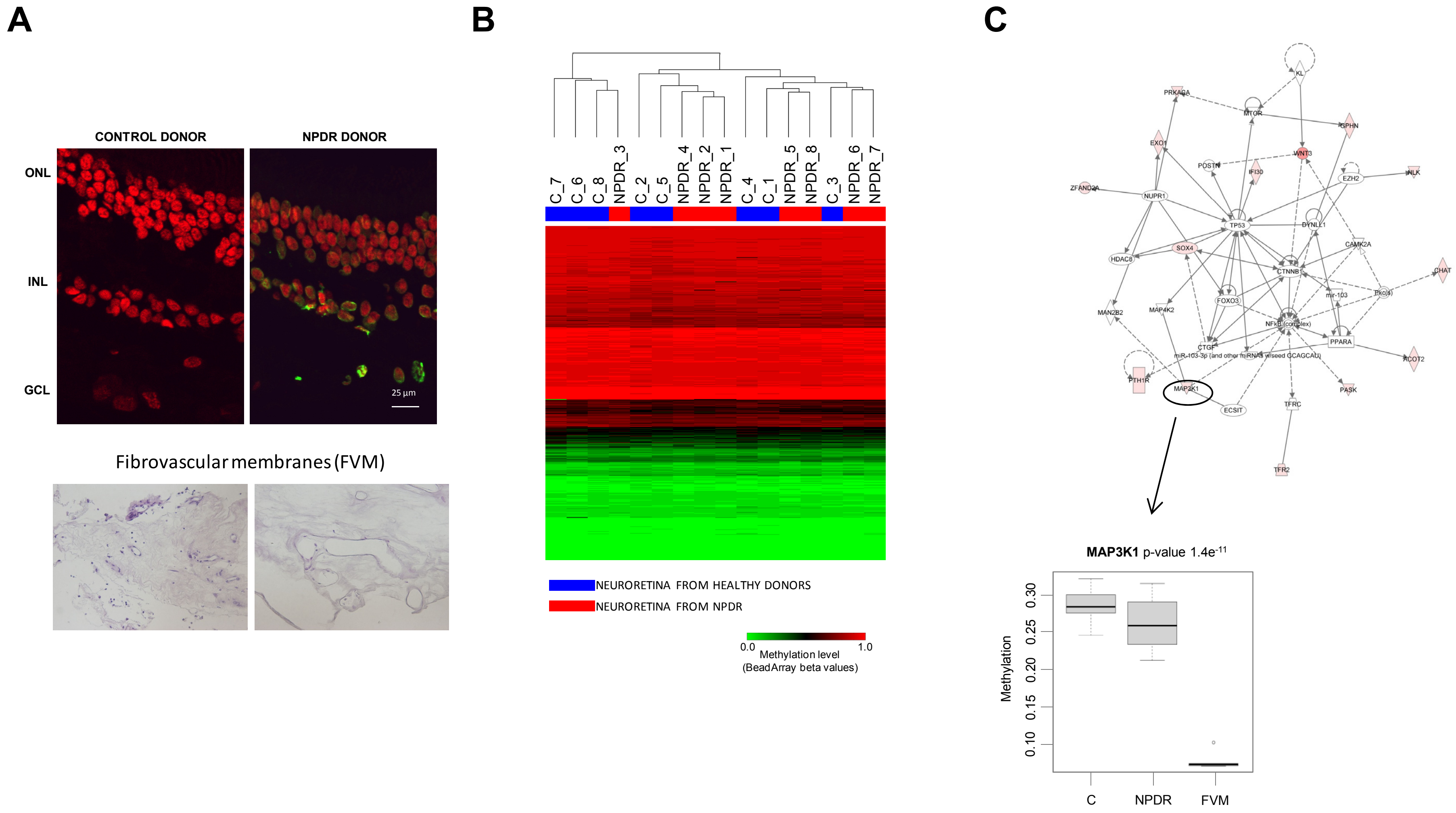

Supplementary Fig. S4

A

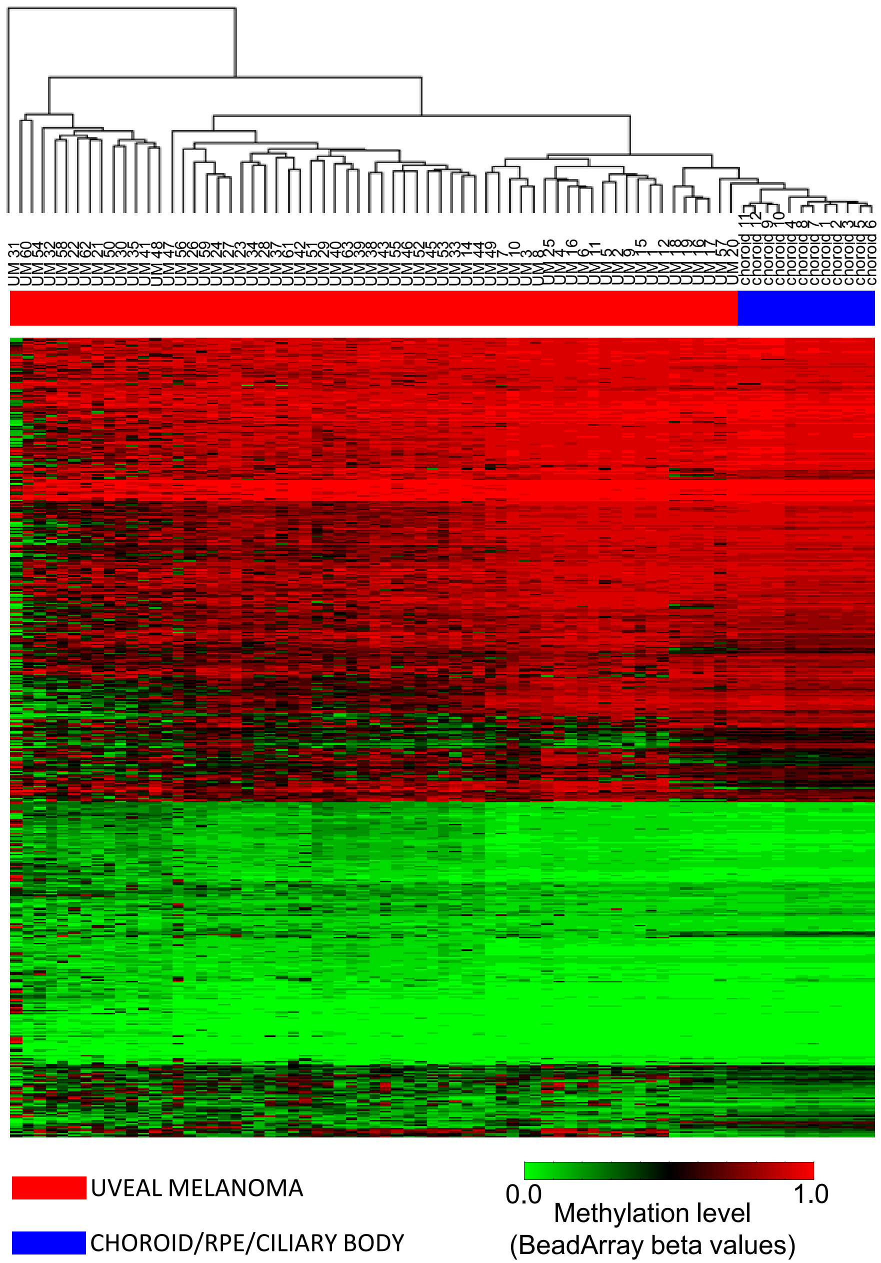

B

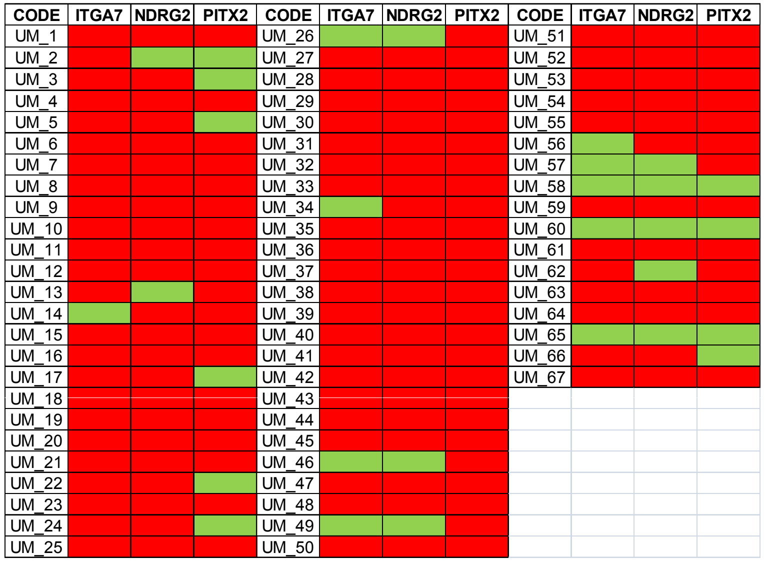

C

ITGA7				
	Goodman and Kruskal's gamma	P-val	N	Kendall b tau
Age		0,1513	63	-0,1800409
Gender	0,08840729	1	67	
Clinical stage	0,05301287	1	63	
Sclera invasion		0,3848	22	0,1857483
Cell type	0,03561157	1	32	
Cilliary body involment	0,05085631	1	56	
Metastasis	0,0840081	0,9999924	32	
Tumor thickness		0,972	54	0,00488503
Largest diameter		0,5202	52	0,08943175

NDRG2				
	Goodman and Kruskal's gamma	P-val	N	Kendall b tau
Age		0,4974	63	0,0856811
Gender	0,08840729		67	
Clinical stage	0,04960663	1	63	
Sclera invasion		0,8513	22	0,0404116
Cell type	0,03732766	0,9999808	32	
Cilliary body involment	0,05510304	1	56	
Metastasis	0,0840081	1	32	
Tumor thickness		0,5207	54	0,08932623
Largest diameter		0,8412	52	0,02791608

PITX2				
	Goodman and Kruskal's gamma	P-val	N	Kendall b tau
Age		0,4044	63	0,1051735
Gender	0,07929137	1	67	
Clinical stage	0,04057492	1	63	
Sclera invasion		0,008662	22	0,5234724
Cell type	0,03732159	0,9999809	32	
Cilliary body involment	0,05510304	1	56	
Metastasis	0,07894737	1	32	
Tumor thickness		0,01047	54	0,3456449
Largest diameter		0,6804	52	0,05735452

Supplementary Fig. S5

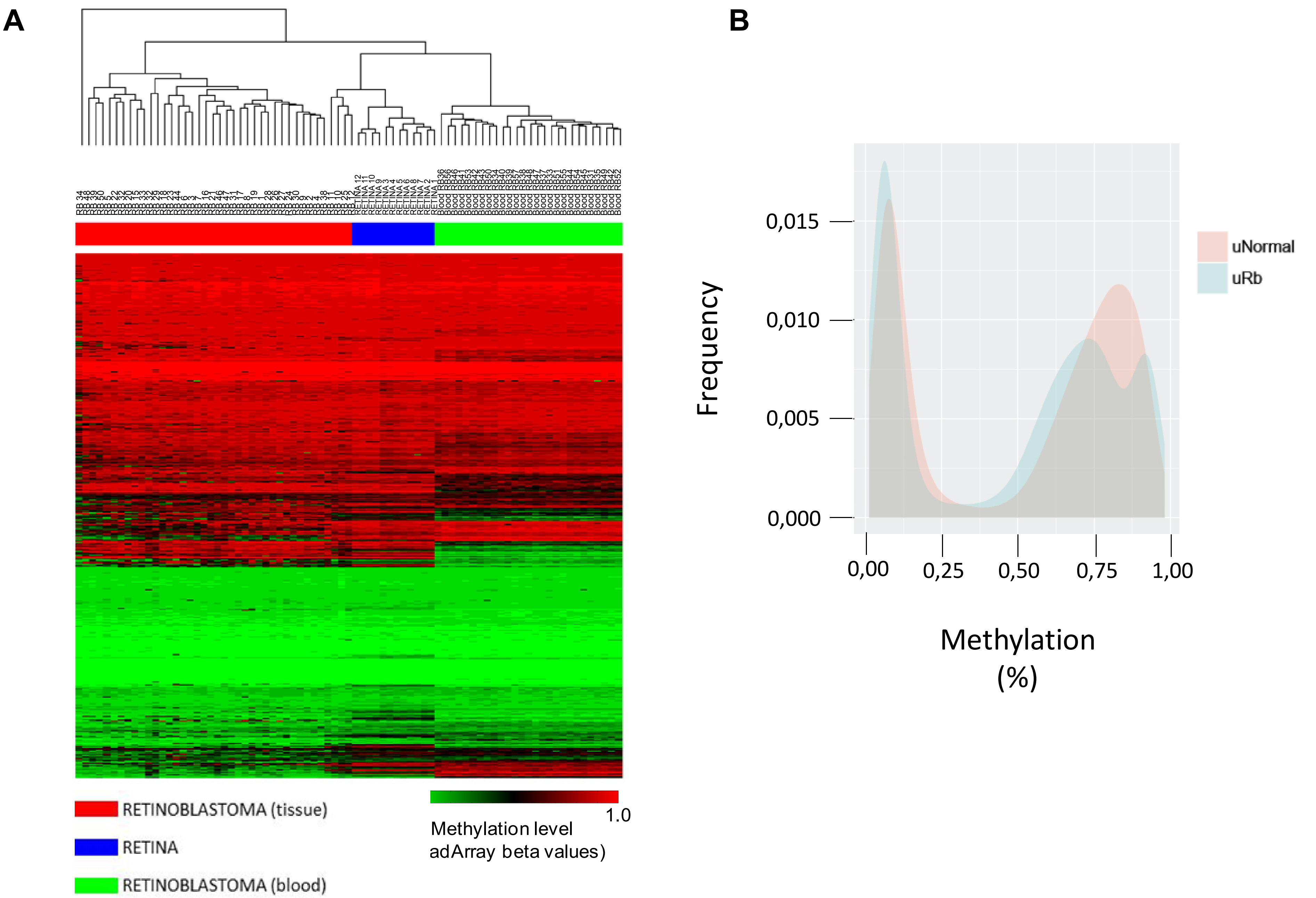

Supplementary Fig. S6

A

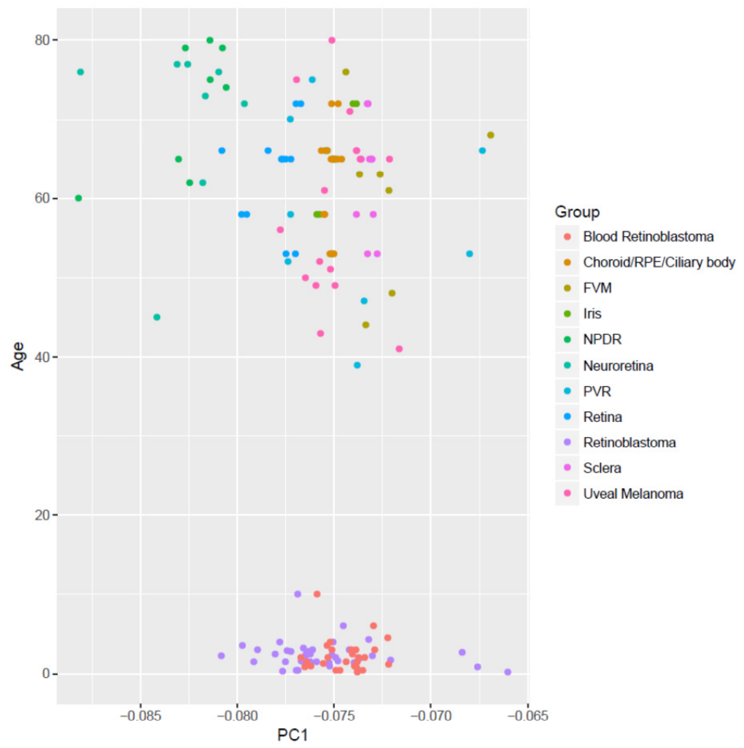

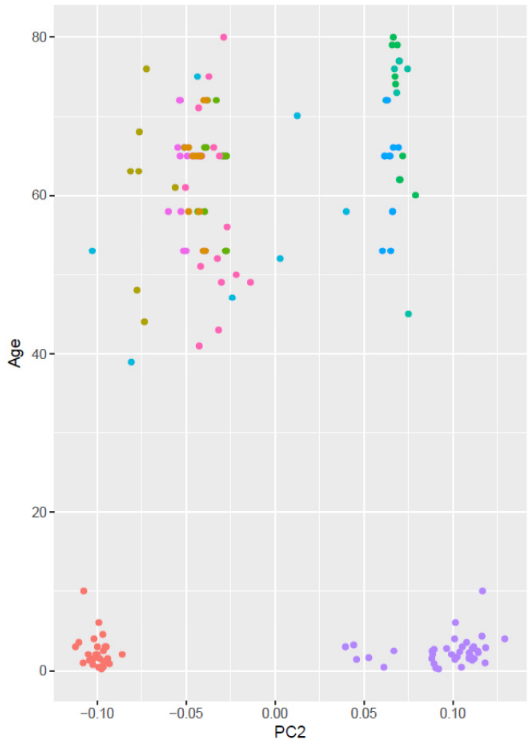

B

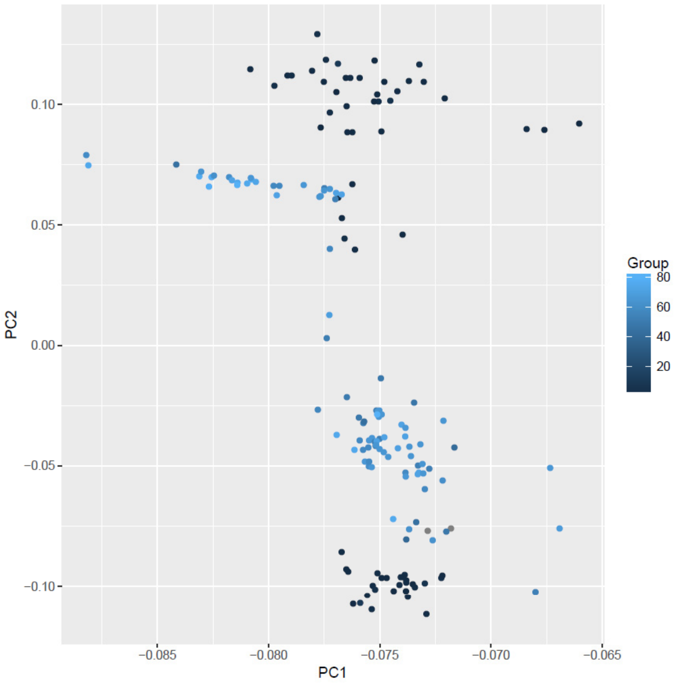

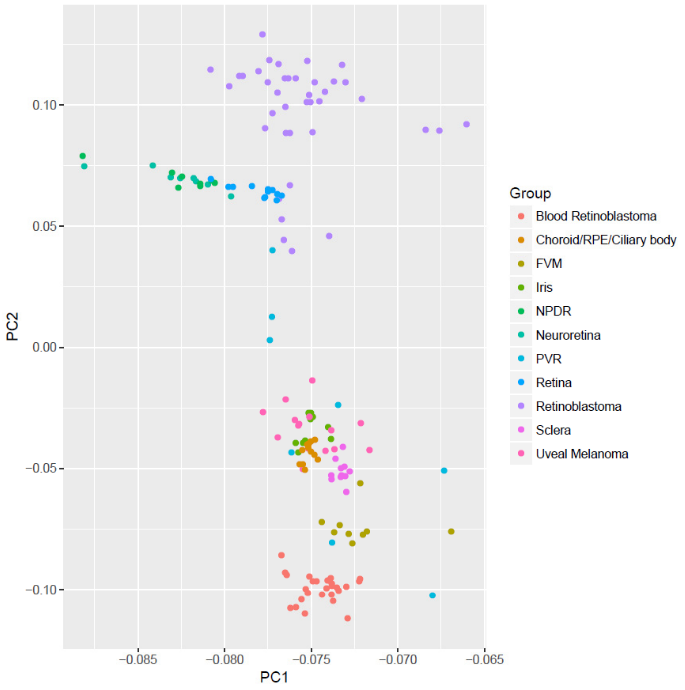

Supplementary Table S1. Clinical features of the human subjects included in the study.

(A) Summary of clinical features of the donors used as normal controls (without ocular diseases).

DONOR	AGE (y)	GENDER	CAUSE OF DEATH	POST-MORTEM TIME	PAST MEDICAL HISTORY
Donor A (eye 1-2)	53	Male	Post-surgery cerebral hemorrhage	3 hours	Hemorrhagic ictus; focal epilepsy; no hypertension; no diabetic; no dyslipidemia
Donor B (eye 3-4)	72	Male	Anoxic post cardiac arrest due to myocardial infarction	3 hours	Prostatitis; tuberculosis (in youth); gastric ulcer
Donor C (eye 5-6)	65	Male	Basal ganglia hemorrhage	4 hours	Hypertension; Chronic renal failure; Peripheral vascular disease
Donor D (eye 7-8)	65	Male	Extensive cerebral hemorrhage	6 hours	Atrial fibrillation; Vertebrobasilar transient ischemic attacks; chronic obstructive pulmonary disease (emphysematous type)
Donor E (eye 9-10)	58	Female	Basilar aneurysmal subarachnoid hemorrhage	6 hours	Migraine, irritable bowel; thyroidectomy for benign thyroid nodule
Donor F (eye 11-12)	66	Female	Respiratory impairment due to drowning	6 hours	Dyslipidemia; osteoporosis

(B) Summary of clinical details of retinal detachment patients (n=8) and *in vitro* cultured fibroblasts (n=2) for studies on Infinium 450K methylation arrays. ERM, epiretinal membrane; GR, globe rupture; PDR, proliferative diabetic retinopathy; PPV, pars plana vitrectomy; PVR, proliferative vitreoretinopathy; RD, retinal detachment; RRD, rhegmatogenous retinal detachment; SubR, subretinal

PATIENT	GENDER	AGE (in years)	RD AETIOLOGY	NUMBER OF PRIOR PROCEDURES	PROCEDURE AT RD SURGERY	PRIOR SILICONE OIL	RETINOTOMY	TIME OF TOTAL RD EVOLUTION (in days)
Infinium 450K methylation arrays								
PVR_1	Male	70	RRD+PVR	4	PPV	Yes	Yes	>90
PVR_2	Male	75	EMR	6	PPV+SubR	Yes	Yes	>90
PVR_3	Male	47	PDR	1	PPV	Yes	Yes	>90
PVR_4	Male	52	GR	5	PPV+SubR	Yes	Yes	>90
PVR_5	Male	39	RRD+PVR	1	PPV	Yes	No	>90
PVR_6	Male	58	RRD+PVR	3	PPV	Yes	Yes	>90
PVR_7	Female	53	RRD+PVR	2	PPV	Yes	Yes	>90
PVR_8	Female	66	RRD+PVR	1	PPV	Yes	Yes	>90
In vitro establishment of fibroblast								
*iv*PVR_01	Female	50	RRD+PVR	1	PPV	No	Yes	60
*iv*PVR_02	PPV	No	Yes	60	PPV	No	Yes	10

(C) Summary of clinical details of diabetic retinopathy patients analyzed by Infinium 450K methylation arrays. Samples from neuroretina from donors without Diabetes mellitus, non-proliferative diabetic retinopathy (NPDR) and fibrovascular membranes (FVM) from diabetic retinopathy patients were included. N.A. Not Applicable; PPV, pars plana vitrectomy; SF6, sulfur hexafluoride.

PATIENT	GENDER	AGE (in years)	Normal/Disease	NUMBER OF PRIOR PROCEDURES	PROCEDURE AT RD SURGERY	PRIOR SILICONE OIL
C_1	Male	62	Normal neuroretina	N.A.	N.A.	N.A.
C_2	Male	77	Normal neuroretina	N.A.	N.A.	N.A.
C_3	Female	73	Normal neuroretina	N.A.	N.A.	N.A.
C_4	Female	76	Normal neuroretina	N.A.	N.A.	N.A.
C_5	Male	76	Normal neuroretina	N.A.	N.A.	N.A.
C_6	Male	72	Normal neuroretina	N.A.	N.A.	N.A.
C_7	Female	77	Normal neuroretina	N.A.	N.A.	N.A.
C_8	Male	45	Normal neuroretina	N.A.	N.A.	N.A.
NPDR_1	Male	62	Diabetes mellitus Type 2	N.A.	N.A.	N.A.
NPDR_2	Male	75	Diabetes mellitus Type 2	N.A.	N.A.	N.A.
NPDR_3	Male	60	Diabetes mellitus Type 2	N.A.	N.A.	N.A.
NPDR_4	Female	65	Diabetes mellitus Type 2	N.A.	N.A.	N.A.
NPDR_5	Female	79	Diabetes mellitus Type 2	N.A.	N.A.	N.A.
NPDR_6	Female	80	Diabetes mellitus Type 2	N.A.	N.A.	N.A.
NPDR_7	Male	79	Diabetes mellitus Type 2	N.A.	N.A.	N.A.
NPDR_8	Male	74	Diabetes mellitus Type 2	N.A.	N.A.	N.A.
FVM_1	Female	68	Diabetes mellitus Type 2	1	PPV+Endolaser	Yes
FVM_2	Female	61	Diabetes mellitus Type 2	1	PPV+Endolaser	No
FVM_3	Male	44	Diabetes mellitus Type 1	5	PPV+Endolaser	Yes
FVM_4	Female	63	Diabetes mellitus Type 2	0	PPV+PPL+Air	No

FVM_5	Female	76	Diabetes mellitus Type 2	0	PPV+PPL+Air	No
FVM_6	Female	48	Diabetes mellitus Type 2	0	PPV+PPL＋SF6	No
FVM_7	Female	63	Diabetes mellitus Type 2	0	PPV+encircling+SF6	No
Pooled samples:						
FVM_8 (Pool 1)	Male	59	Diabetes mellitus Type 2	1	PPV+SF6	No
FVM_9 (Pool 1)	Male	60	Diabetes mellitus Type 2	0	PPV+PPL+Air	No
FVM_10 (Pool 2)	Male	45	Diabetes mellitus Type 2	0	PPV+SF6	No
FVM_11 (Pool 2)	Male	49	Diabetes mellitus Type 2	0	PPV+PPL+Air	No
FVM_12 (Pool 2)	Male	46	Diabetes mellitus Type 2	0	PPV+PPL	No

(D) Summary of clinical, histopathologic and molecular data of uveal melanoma samples analyzed by Infinium 450K methylation arrays (n=63) and the independent cohort for results' validation (n=67).

[1] Tumor size/Lymph Node involvement/Metastasis (TNM) Classification of Malignant Tumours by the International Union Against Cancer

[2] Anatomic Stage according to American Joint Committee on Cancer Guidelines.

[3] For mixed populations the ratios of Epitheloid/Spindle cells are provided.

SAMPLE	AGE (y)	GENDER	CILIARY BODY INVOLVEMENT	TNM[1]	STAGE[2]	SCLERAL INVASION	CELL TYPE[3]	MITOSIS	NECROSIS (%)	METASTASIS
Infinium methylation arrays:										
MEL_1	49	Male	No	T3aN0M0	IIB	N/ A	N/ A	N/ A	N/ A	No
MEL_2	52	Female	Yes	T3aN0M0	IIB	Intrascleral	Mixed (80/20)	3/50 fields	0	No
MEL_3	71	Male	No	T4aN0M0	IIIA	Extrascleral localized	Epithelioid	5/20 fields	60	Yes
MEL_4	50	Female	No	T1aN0M0	I	Intrascleral localized	Mixed (80/20)	13/50 fields	0	No
MEL_5	66	Female	No	T2aN0M0	IIA	None	Spindle	3/50 fields	0	No
MEL_6	43	Male	No	T4eN0M0	IIIC	Extrascleral diffuse	Mixed (50/50)	3/20 fields	20	No
MEL_7	41	Male	No	T1aN0M0	I	None	Spindle	1/50 fields	35	No
MEL_8	51	Female	No	T3aN0M0	IIB	None	Epithelioid	3/50 fields	10	Yes
MEL_9	61	Male	No	T3aN0M0	IIB	N/ A	N/ A	N/ A	N/ A	No
MEL_10	49	Female	No	T4eN0M0	IIIC	Extrascleral localized	Mixed (80/20)	14/50 fields	10	No
MEL_11	56	Female	Yes	T4aN0M0	IIIA	Extrascleral localized	Mixed (20/80)	2/50 fields	20	No
MEL_12	75	Female	No	T4bN0M0	IIIB	Intrascleral localized	Mixed (50/50)	13/20 fields	40	No
MEL_13	65	Female	Yes	T4aN0M0	IIIA	Intrascleral	Epithelioid	19/50 fields	40	Yes
MEL_14	65	Female	No	T1aN0M0	I	None	Epithelioid	4/20 fields	0	Yes

MEL_15	80	Male	No	T3aN0M0	IIB	Intrascleral	Mixed (80/20)	4/50 fields	0	No
MEL_16	N/ A	N/ A	N/ A	N/ A	N/ A	N/ A	N/ A	N/ A	N/ A	N/ A
MEL_17	N/ A	N/ A	N/ A	N/ A	N/ A	N/ A	N/ A	N/ A	N/ A	N/ A
MEL_18	N/ A	N/ A	N/ A	N/ A	N/ A	N/ A	N/ A	N/ A	N/ A	N/ A
MEL_19	N/ A	N/ A	N/ A	N/ A	N/ A	N/ A	N/ A	N/ A	N/ A	N/ A
MEL_20	N/ A	N/ A	N/ A	N/ A	N/ A	N/ A	N/ A	N/ A	N/ A	N/ A
MEL_21	74	Male	N/ A	N/ A	N/ A	N/ A	N/ A	N/ A	N/ A	N/ A
MEL_22	40	Male	N/ A	N/ A	N/ A	N/ A	N/ A	N/ A	N/ A	N/ A
MEL_23	N/ A	N/ A	N/ A	N/ A	N/ A	N/ A	N/ A	N/ A	N/ A	N/ A
MEL_24	71	Male	N/ A	N/ A	N/ A	N/ A	N/ A	N/ A	N/ A	N/ A
MEL_25	N/ A	N/ A	N/ A	N/ A	N/ A	N/ A	N/ A	N/ A	N/ A	N/ A
MEL_26	13	Female	N/ A	N/ A	N/ A	N/ A	N/ A	N/ A	N/ A	N/ A
MEL_27	42	Female	N/ A	N/ A	N/ A	N/ A	N/ A	N/ A	N/ A	N/ A
MEL_28	N/ A	N/ A	N/ A	N/ A	N/ A	N/ A	N/ A	N/ A	N/ A	N/ A
MEL_29	44	Male	N/ A	N/ A	N/ A	N/ A	Spindle	N/ A	N/ A	N/ A
MEL_30	55	Male	N/ A	N/ A	N/ A	N/ A	N/ A	N/ A	N/ A	N/ A
MEL_31	37	Male	N/ A	N/ A	N/ A	N/ A	Epithelioid	N/ A	N/ A	N/ A
MEL_32	57	Male	N/ A	N/ A	N/ A	N/ A	N/ A	N/ A	N/ A	N/ A
MEL_33	58	Male	N/ A	N/ A	N/ A	N/ A	Epithelioid	N/ A	N/ A	N/ A
MEL_34	N/ A	N/ A	N/ A	N/ A	N/ A	N/ A	N/ A	N/ A	N/ A	N/ A
MEL_35	60	Male	N/ A	N/ A	N/ A	N/ A	N/ A	N/ A	N/ A	N/ A
MEL_36	40	Male	N/ A	N/ A	N/ A	N/ A	Spindle	N/ A	N/ A	N/ A
MEL_37	47	Male	N/ A	N/ A	N/ A	N/ A	N/ A	N/ A	N/ A	N/ A
MEL_38	N/ A	N/ A	N/ A	N/ A	N/ A	N/ A	N/ A	N/ A	N/ A	N/ A
MEL_39	54	Female	N/ A	N/ A	N/ A	N/ A	N/ A	N/ A	N/ A	N/ A
MEL_40	56	Female	N/ A	N/ A	N/ A	N/ A	N/ A	N/ A	N/ A	N/ A
MEL_41	35	Female	N/ A	N/ A	N/ A	N/ A	N/ A	N/ A	N/ A	N/ A
MEL_42	58	Male	N/ A	N/ A	N/ A	N/ A	Spindle	N/ A	N/ A	N/ A
MEL_43	32	Female	N/ A	N/ A	N/ A	N/ A	Spindle	N/ A	N/ A	N/ A
MEL_44	60	Male	N/ A	N/ A	N/ A	N/ A	N/ A	N/ A	N/ A	N/ A
MEL_45	63	Female	N/ A	N/ A	N/ A	N/ A	N/ A	N/ A	N/ A	N/ A
MEL_46	35	Male	N/ A	N/ A	N/ A	N/ A	N/ A	N/ A	N/ A	N/ A
MEL_47	51	Male	N/ A	N/ A	N/ A	N/ A	Spindle	N/ A	N/ A	N/ A
MEL_48	51	Female	N/ A	N/ A	N/ A	N/ A	Epithelioid	N/ A	N/ A	N/ A
MEL_49	58	Female	N/ A	N/ A	N/ A	N/ A	N/ A	N/ A	N/ A	N/ A

MEL_50	55	Male	N/ A	N/ A	N/ A	N/ A	N/ A	N/ A	N/ A	N/ A
MEL_51	N/ A	N/ A	N/ A	N/ A	N/ A	N/ A	N/ A	N/ A	N/ A	N/ A
MEL_52	48	Male	N/ A	N/ A	N/ A	N/ A	N/ A	N/ A	N/ A	N/ A
MEL_53	37	Female	N/ A	N/ A	N/ A	N/ A	N/ A	N/ A	N/ A	N/ A
MEL_54	69	Male	N/ A	N/ A	N/ A	N/ A	Mixed	N/ A	N/ A	N/ A
MEL_55	71	Male	N/ A	N/ A	N/ A	N/ A	Spindle	N/ A	N/ A	N/ A
MEL_56	57	Female	N/ A	N/ A	N/ A	N/ A	Epithelioid	N/ A	N/ A	N/ A
MEL_57	42	Male	N/ A	N/ A	N/ A	N/ A	Epithelioid	N/ A	N/ A	N/ A
MEL_58	N/ A	N/ A	N/ A	N/ A	N/ A	N/ A	N/ A	N/ A	N/ A	N/ A
MEL_59	64	Female	N/ A	N/ A	N/ A	N/ A	N/ A	N/ A	N/ A	N/ A
MEL_60	N/ A	N/ A	N/ A	N/ A	N/ A	N/ A	N/ A	N/ A	N/ A	N/ A
MEL_61	N/ A	N/ A	N/ A	N/ A	N/ A	N/ A	N/ A	N/ A	N/ A	N/ A
MEL_62	58	Male	N/ A	N/ A	N/ A	N/ A	Epithelioid	N/ A	N/ A	N/ A
MEL_63	62	Male	N/ A	N/ A	N/ A	N/ A	Spindle	N/ A	N/ A	N/ A
Validation cohort:										
UV_1	53	Male	N/A	N/A	N/A	N/A	N/A	N/A	N/A	Yes
UV_2	76	Female	No	T2aN0M0	IIA	None	N/A	N/A	N/A	No
UV_3	58	Female	No	T2aN0M0	IIA	None	N/A	N/A	N/A	No
UV_4	71	Female	No	T3aN0M0	IIB	None	N/A	N/A	N/A	No
UV_5	39	Male	No	T2aN0M0	IIA	None	N/A	N/A	N/A	No
UV_6	56	Male	No	T4aN0M0	IIIA	None	N/A	N/A	N/A	Yes
UV_7	38	Female	No	T4aN0M0	IIIA	None	N/A	N/A	N/A	No
UV_8		Female	N/A	N/A	N/A	N/A	N/A	N/A	N/A	No
UV_9	31	Male	No	T3aN0M0	IIB	None	N/A	N/A	N/A	Yes
UV_10	71	Male	No	T4aN0M0	IIIA	None	N/A	N/A	N/A	Yes
UV_11	72	Male	No	T4aN0M0	IIIA	None	N/A	N/A	N/A	Yes
UV_12	56	Male	No	T3bN0M0	IIIA	None	N/A	N/A	N/A	No
UV_13	35	Male	No	T3aN0M0	IIB	None	N/A	N/A	N/A	No
UV_14		Male	N/A	N/A	N/A	N/A	N/A	N/A	N/A	No
UV_15	82	Female	No	T3aN0M0	IIB	None	N/A	N/A	N/A	No
UV_16	63	Female	No	T4aN0M0	IIIA	N/A	N/A	N/A	N/A	No
UV_17	73	Female	No	T4aN0M0	IIIA	Intrascleral	Mixed (80/20)	4/50 fields	<10	No
UV_18	70	Female	No	T3aN0M0	IIB	None	N/A	N/A	N/A	Yes
UV_19	85	Male	No	T4aN0M0	IIIA	Intrascleral	Mixed (80/20)	0,5/50 fields	No	Yes

UV_20	66	Female	No	T3aN0M0	IIB	None	Mixed (20/80)	1/50 fields	10-50	Yes
UV_21	45	Female	No	T1cN0M0	IIA	Extrascleral diffuse	Mixed (60/40)	1/50 fields	No	No
UV_22	71	Male	No	T2aN0M0	IIA	None	N/A	N/A	N/A	No
UV_23	70	Female	No	T3bN0M0	IIIA	None	N/A	N/A	N/A	No
UV_24	81	Male	No	T3cN0M0	IIIA	Intrascleral	N/A	N/A	N/A	Yes
UV_25	54	Male	No	T4aN0M0	IIIA	Intrascleral	epithelioid	26/50 fields	<10	Yes
UV_26	76	Male	No	T4bN0M0	IIIB	Intrascleral	N/A	N/A	>50	Yes
UV_27	46	Male	Yes	T4bN0M0	IIIB	N/A	N/A	N/A	N/A	Yes
UV_28	42	Female	No	T3aN0M0	IIA	Intrascleral	Mixed (70/30)	7/50 fields	No	No
UV_29	76	Male	No	T3aN0M0	IIA	N/A	N/A	N/A	N/A	No
UV_30	68	Female	Yes	T4bN0M0	IIIB	Intrascleral	Mixed (40/60)	3/50 fields	10-50	Yes
UV_31	56	Male	No	T2cN0M0	IIIA	Intrascleral	Epithelioid	1/50 fields	<10	No
UV_32	61	Male	No	T4aN0M0	IIIA	N/A	N/A	N/A	N/A	Yes
UV_33	80	Male	No	T4cN0M0	IIIB	Extrascleral localized	Mixed (70/30)	10/50 fields	<10	Yes
UV_34	72	Male	No	T4aN0M0	IIIA	N/A	N/A	N/A	N/A	No
UV_35	80	Female	No	T3aN0M0	IIB	N/A	N/A	N/A	N/A	No
UV_36	51	Female	Yes	T4aN0M0	IIIA	N/A	N/A	N/A	N/A	No
UV_37	83	Male	No	T4aN0M0	IIIA	Intrascleral	Mixed (30/70)	1/50 fields	No	Yes
UV_38	74	Male	No	T4bN0M0	IIIB	Intrascleral	Mixed (40/60)	8/50 fields	<10	No
UV_39	32	Female	No	T3cN0M0	IIIA	Extrascleral localized	Spindle	4/40 fields	No	No
UV_40	39	Female	No	T4aN0M0	IIIA	Intrascleral	Mixed (30/70)	13/50 fields	10-50	No
UV_41	29	Female	No	T3aN0M0	IIB	none	Spindle	15/50 fields	No	No
UV_42	63	Female	No	T3aN0M0	IIB	none	Epithelioid	20/50 fields	No	Yes

UV_43	63	Female	No	T3aN0M0	IIB	none	Epithelioid	5/50 fields	No	No
UV_44	79	Female	No	T4bN0M0	IIIB	N/A	N/A	N/A	N/A	No
UV_45	72	Male	No	T3cN0M0	IIIA	intrascleral	Epithelioid	8/50 fields	No	No
UV_46	62	Male	No	T4aN0M0	IIIA	N/A	N/A	N/A	N/A	Yes
UV_47	67	Female	No	T3aN0M0	IIB	N/A	N/A	N/A	N/A	No
UV_48	67	Female	No	T4aN0M0	IIIA	N/A	N/A	N/A	N/A	No
UV_49	61	Male	No	T3aN0M0	IIB	N/A	N/A	N/A	N/A	Yes
UV_50	80	Male	No	T4aN0M0	IIIA	none	Spindle	7/50 fields	10-50	No
UV_51	61	Male	No	T4aN0M0	IIIA	Intrascleral	Mixed	9/50 fields	<10	No
UV_52	76	Male	No	T3aN0M0	IIB	Intrascleral	Epitheliolid	5/50 fields	10-50	Yes
UV_53	45	Male	No	T3bN0M0	IIIA	Intrascleral	Spindle	16/50 fields	<10	No
UV_54	66	Male	No	T4bN0M0	IIIB	Extrascleral diffuse	Epithelioid	N/A	N/A	Yes
UV_55	83	Female	No	T4aN0M0	IIIA	N/A	Mixed (70/30)	N/A	N/A	No
UV_56	62	Female	No	T2aN0M0	IIA	intrascleral	Epithelioid	N/A	<10	No
UV_57	73	Female	N/A	T4aN0M0	IIIA	N/A	N/A	N/A	N/A	Yes
UV_58	59	Female	N/A	T4aN0M0	IIIA	N/A	N/A	N/A	N/A	Yes
UV_59	38	Female	No	T1aN0M0	I	N/A	Epithelioid	N/A	N/A	No
UV_60	90	Female	N/A	T4aN0M0	IIIA	N/A	N/A	N/A	N/A	No
UV_61	39	Female	N/A	N/A	N/A	N/A	N/A	N/A	N/A	No
UV_62	54	Male	No	T3aN0M0	IIB	intrascleral	Spindle	N/A	<10	No
UV_63	68	Male	No	T2aN0M0	IIA	N/A	N/A	N/A	N/A	No
UV_64	76	Male	N/A	T2aN0M0	IIA	N/A	N/A	N/A	N/A	No
UV_65	66	Male	N/A	T2aN0M0	IIA	N/A	N/A	N/A	N/A	No
UV_66	46	Female	N/A	T2aN0M0	IIA	N/A	N/A	N/A	N/A	No
UV_67	41	Male	N/A	T4aN0M0	IIIA	N/A	N/A	N/A	N/A	No

(E) Summary of clinical, histopathological and molecular data of retinoblastoma patients.

[1] International retinoblastoma stage (*Murphree AL. 2005. Ophthalmol Clin North Am 18:41-53)*

[2] Retinoblastoma Classification according to Reese–Ellsworth Classification for Conservative Treatment of Retinoblastoma Guideline.

[3] LE, left eye; RE, right eye; OU: both eyes

[4] Anterior chamber (AC), Iris (I), choroidal (Ch) and subretinal (SR) invasion was scored for each sample.

[5] The presence of clinically visible tumor seeds was defined as: Vitreal (V), Subretinal (SR) or absence of tumor seeds (No).

PATIENT	GENDER	AGE (MONTHS)	STAGE R-E[2]	EYES AFFECTED[3]	HISTOLOGY	GROWTH	INVASION [4]	OPTIC NERVE	SEEDS[5]	RB1 MUTATION (in blood)
RB_1	Male	51	5A	LE	Undifferentiated	Mixed	Ch (focal), SR	Laminar	V, SR	Normal
RB_2	Male	48	5B-2	LE	Moderately differentiated	Endophytic	SR	Non affected	V, SR	Normal
RB_3	Male	29	5A	LE	Undifferentiated	Mixed	Ch (focal), SR, AC, I	Surgical margin	V, SR	Normal
RB_4	Male	17	5B-1	LE	Moderately differentiated	Mixed	Ch, SR	Laminar	No	Normal
RB_5	Female	21	5A	RE	Moderately differentiated	Mixed	Ch, SR	Laminar	No	Normal
RB_6	Male	16	5A	LE	Moderately differentated	Mixed	Ch (focal)	Non affected	V, SR	Normal
RB_7	Female	33	5A	RE	Differentated	Mixed	Ch (focal)	Non affected	SR	Normal
RB_8	Female	19	5B-1	LE	Moderately differentiated	Mixed	Ch, SR	Laminar	No	Normal
RB_9	Male	34	5B	LE	Undifferentiated	Mixed	Ch (focal), SR	Non affected	V, SR	Normal
RB_10	Male	39	5B-2	RE	Moderately differentiated	Mixed	Ch (focal), SR	Non affected	SR	Normal
RB_11	Female	4	5B-1	AU	Differentiated	Endophytic	Ch (focal)	Non affected	V	c.381-1G>T
RB_12	Male	19	4A	AU	Differentiated	Mixed	SR	Non affected	No	c.43_80del
RB_13	Female	48	5B	RE	N/A	Mixed	No	Non Affected	V	g.150091C>T
RB_14	Male	2	N/A	RE; LE	N/A	N/A	N/A	N/A	N/A	N/A

RB_15	Male	30	N/A	LE	N/A	N/A	N/A	N/A	N/A	NO
RB_16	Male	42	N/A	RE	N/A	N/A	N/A	N/A	N/A	N/A
RB_17	Female	23	N/A	LE	N/A	N/A	N/A	N/A	N/A	N/A
RB_18	Male	18	N/A	LE	N/A	N/A	N/A	N/A	N/A	NO
RB_19	Female	11	N/A	RE	N/A	N/A	N/A	N/A	N/A	g.162237C>T
RB_20	Male	17	N/A	LE	N/A	N/A	N/A	N/A	N/A	NO
RB_21	Male	36	N/A	LE	N/A	N/A	N/A	N/A	N/A	g.156713C>T
RB_22	Male	32	N/A	RE	N/A	N/A	N/A	N/A	N/A	NO
RB_23	Female	27	N/A	RE; LE	N/A	N/A	N/A	N/A	N/A	g.65435_6delCA
RB_24	Female	28	N/A	RE; LE	N/A	N/A	N/A	N/A	N/A	Gross deletion
RB_25	Male	36	N/A	LE	N/A	N/A	N/A	N/A	N/A	NO
RB_26	Male	35	N/A	RE	N/A	N/A	N/A	N/A	N/A	NO
RB_27	Male	36	N/A	RE	N/A	N/A	N/A	N/A	N/A	NO
RB_28	Female	27	N/A	RE; LE	N/A	N/A	N/A	N/A	N/A	Gross deletion
RB_29	Male	48	4B	OU	Undifferentiated	Mixed	AC, SR	Pre-laminar	SR	Normal
RB_30	Male	30	5B	RE	Moderately differentiated	Mixed	AC, SR	Laminar	V, SR	Normal
RB_31	Female	72	5A	RE	Undifferentiated	Mixed	SR	Non affected	V	N/A
RB_32	Male	2	5A	LE	Undifferentiated	Mixed	ch	Non affected	NO	N/A
RB_33	Female	18	5A	LE	Moderately differentiated	Mixed	CH	Non affected	V	N/A
RB_34	Male	10	5B	LE	Moderately differentiated	Mixed	AC, CH	Non affected	V	N/A
RB_35	Female	9	5A	RE	Differentiated	Endophytic	CH	Non affected	SR	N/A
RB_36	Female	36	5A	LE	Differenciated	mixed	SR	Non affected	V	N/A
RB_37	Female	36	5A	LE	Differenciated	mixed	CH	Laminar	V	N/A
RB_38	Male	18	5B	RE	Moderately differentiated	Endophytic	CH, AC, I,SR	Non affected	SR,V	N/A
RB_39	Male	24	5A	RE	Moderately	Mixed	AC, I, CH	Laminar	V	N/A

					differentiated					
RB_40	Male	24	5A	OU	Differentiated	Mixed	CH	Non affected	V	N/A
RB_41	Male	54	5A	RE	Undifferenciated	Mixed	CH	Laminar	V	N/A
RB_42	Male	5	5A	LE	Moderately differentiated	Mixed	CH	Non affected	SR	N/A
RB_43	Male	14	5A	RE	Differenciated	Mixed	CH	Laminar	V	N/A
RB_44	Male	120	5A	LE	Moderately differentiated	Mixed	CH	Laminar	NO	N/A
RB_45	Female	12	5A	RE	Undifferenciated	Endophytic	SR	Non affected	V	N/A
RB_46	Female	5	5A	OU	Differentiated	Mixed	CH	Non affected	V	N/A
RB_47	Female	18	5A	LE	Undifferenciated	Mixed	CH	Non affected	SR	N/A
RB_48	Male	24	5A	OU	Moderately differentiated	Exophytic	AC, CH,SR,I	Laminar	SR,V	N/A
RB_49	Female	36	5A	ou	Undifferenciated	Mixed	AC	Laminar	NO	N/A
RB_50	Female	5	5A	OU	Undifferentiated	Mixed	CH	Non affected	V	N/A
RB_51	Male	30	5A	RE	Differentiated	Mixed	CH,AC	Laminar	SR	N/A
RB_52	Male	36	5A	RE	Differenciated	Mixed	CH	Non affected	SR	N/A
RB_53	Male	15	5B	RE	Moderately differentiated	Mixed	CH	Non affected	V	N/A
RB_54	Male	42	5B	RE	Differenciated	Mixed	CH,SR,AC,I	Laminar	V	N/A
RB_55	Male	24	5B	OU	Differentiated	Mixed	CH	Non affected	V	N/A
RB_56	Female	11	5B	OU	Differentiated	Mixed	AC	Laminar	V	N/A
RB_57	Male	48	5B	LE	Moderately differentiated	Mixed	CH	Non affected	SR	N/A

Supplementary Table S2. List of retinal genes showing differential methylation among retina and the remaining eye layers (n=24). Categorization as retinal genes was performed according to The Tissue-Specific Genes Database (TISGED).

Gene Symbol	Gene ID	Function	Methylation status in retina
FAM83A	84985	family with sequence similarity 83, member A	Hypermethylated
IFITM1	8519	interferon induced transmembrane protein 1	Hypermethylated
MMEL1	79258	membrane metallo-endopeptidase-like 1	Hypermethylated
TMPRSS13	84000	transmembrane protease, serine 13	Hypermethylated
VIT	5212	vitrin	Hypermethylated
AIPL1	23746	aryl hydrocarbon receptor interacting protein-like 1	Hypomethylated
CABP4	57010	calcium binding protein 4	Hypomethylated
CNGA1	1259	cyclic nucleotide gated channel alpha 1	Hypomethylated
CRX	1406	cone-rod homeobox	Hypomethylated
GNAT1	2779	guanine nucleotide binding protein (G protein), alpha transducing activity polypeptide 1	Hypomethylated
GNB5	10681	guanine nucleotide binding protein (G protein), beta 5	Hypomethylated
GRK1	6011	G protein-coupled receptor kinase 1	Hypomethylated
GUCA1B	2979	guanylate cyclase activator 1B (retina)	Hypomethylated
IMPG2	50939	interphotoreceptor matrix proteoglycan 2	Hypomethylated
KCNB1	3745	potassium voltage-gated channel, Shab-related subfamily, member 1	Hypomethylated
LRIT1	26103	leucine-rich repeat, immunoglobulin-like and transmembrane domains 1	Hypomethylated
LRIT2	340745	leucine-rich repeat, immunoglobulin-like and transmembrane domains 1	Hypomethylated
MARCH1	55016	membrane-associated ring finger (C3HC4) 1, E3 ubiquitin protein ligase	Hypomethylated
PCMTD2	55251	protein-L-isoaspartate (D-aspartate) O-methyltransferase domain containing 2	Hypomethylated
PDE6A	5145	phosphodiesterase 6A, cGMP-specific, rod, alpha	Hypomethylated
PDE6G	5148	phosphodiesterase 6G, cGMP-specific, rod, gamma	Hypomethylated
PPEF2	5470	protein phosphatase, EF-hand calcium binding domain 2	Hypomethylated
SLC24A1	9187	solute carrier family 24 (sodium/potassium/calcium exchanger), member 1	Hypomethylated
TULP1	7287	tubby like protein 1	Hypomethylated

Supplementary Table S3. β- methylation values obtained for 42 representative well-studied DNA- binding transcription factors involved in ocular tissues development (reviewed in [2]) in retina, sclera, iris and choroid/RPE/ciliary body. Absolute average (AVG) methylation values were calculated for each tissue. ret-CpGs were calculated as the average in retina samples (AVG_retina) minus the average of the remaining tissues ((AVG_ (choroid/RPE/ciliary body + iris + sclera)).

TargetID	AVG_RETINA	AVG_(CHOROID/RPE/CILIARY BODY)+IRIS+SCLERA	ret_CpGs	UCSC_REFGENE_NAME
cg27189087	0,704103358	0,098131768	0,60597159	RAX
cg16071219	0,790968933	0,291316034	0,4996529	LPAR6;LPAR6;RB1;LPAR6
cg26932432	0,651830258	0,174648228	0,47718203	PAX6;PAX6;PAX6
cg09750084	0,76464745	0,295332524	0,46931493	LPAR6;RB1
cg03646329	0,822139325	0,3590665	0,46307283	LPAR6;LPAR6;LPAR6;RB1;LPAR6
cg09656389	0,6455717	0,183824157	0,46174754	PAX6
cg01298678	0,892600217	0,434384291	0,45821593	LHX2
cg14439629	0,67810775	0,222867071	0,45524068	PAX6;PAX6;PAX6
cg21195468	0,757506383	0,303843277	0,45366311	LHX2
cg07573727	0,671802825	0,22321671	0,44858612	VSX2;VSX2
cg23095743	0,618620392	0,189607031	0,42901336	PITX3
cg20327163	0,682222917	0,256382134	0,42584078	NR2F2;NR2F2;NR2F2;NR2F2
cg09217215	0,644720267	0,23139068	0,41332959	PAX6;PAX6;PAX6
cg19006378	0,587053708	0,181142878	0,40591083	PAX6;PAX6;PAX6
cg03671802	0,708901742	0,306924803	0,40197694	RAX
cg15416329	0,711844225	0,312782306	0,39906192	VSX2
cg12160586	0,69726815	0,29916356	0,39810459	ASCL1
cg06620569	0,726645133	0,333472166	0,39317297	RAX
cg20023259	0,586826917	0,196964506	0,38986241	PITX3
cg18503031	0,875953433	0,490601654	0,38535178	MITF;MITF;MITF
cg03831180	0,626675967	0,24230407	0,3843719	MITF;MITF;MITF
cg04504066	0,522482183	0,141590245	0,38089194	PAX6;PAX6;PAX6

cg02468250	0,626729025	0,246006243	0,38072278	PAX6;PAX6;PAX6
cg06808498	0,614094233	0,238528726	0,37556551	SIX6
cg00814752	0,616638842	0,246754549	0,36988429	RAX
cg19764143	0,599396425	0,233556966	0,36583946	SIX6
cg09041678	0,6179743	0,25618852	0,36178578	PAX6
cg08675869	0,445162108	0,102788677	0,34237343	NR2F2;NR2F2;NR2F2;NR2F2;NR2F2
cg11482099	0,460571208	0,129243075	0,33132813	PAX6;PAX6;PAX6
cg13523819	0,887901767	0,558732754	0,32916901	MITF;MITF;MITF
cg23734137	0,835448342	0,5086505	0,32679784	NR2F2
cg01867395	0,516922083	0,190674418	0,32624767	PAX6
cg05555207	0,508631458	0,1859133	0,32271816	TBX5;TBX5
cg08318726	0,536597975	0,215319628	0,32127835	TBX5;TBX5;TBX5
cg12279294	0,602085092	0,282881417	0,31920367	NR2F2;NR2F2;NR2F2;NR2F2
cg07434271	0,542797533	0,224013386	0,31878415	PAX6
cg06312283	0,579773942	0,26192332	0,31785062	PAX6
cg06911121	0,444420142	0,130432528	0,31398761	TBX5;TBX5
cg25242557	0,457745833	0,144181715	0,31356412	PAX6;PAX6;PAX6
cg21611810	0,583444942	0,279636206	0,30380874	TBX5;TBX5;TBX5;TBX5
cg19592637	0,722365108	0,418705089	0,30366002	VSX1;VSX1
cg11841394	0,571093608	0,268883863	0,30220975	TBX5;TBX5;TBX5;TBX5
cg20099830	0,661297075	0,360253497	0,30104358	TBX5;TBX5;TBX5;TBX5
cg09816693	0,654029375	0,356352429	0,29767695	VSX2
cg12086936	0,394792342	0,099983802	0,29480854	PAX6;PAX6;PAX6
cg13570972	0,4612236	0,168616034	0,29260757	PAX6
cg17280740	0,704187567	0,41343984	0,29074773	PAX6
cg01665555	0,512181825	0,223842829	0,288339	PAX6;PAX6;PAX6
cg12827555	0,638573867	0,354839903	0,28373396	VSX2
cg23118730	0,51731745	0,234065723	0,28325173	VSX2

cg22272457	0,4337396	0,153359865	0,28037973	PAX6;PAX6;PAX6
cg13151171	0,784489033	0,504448197	0,28004084	MITF;MITF;MITF
cg05929882	0,4665303	0,198842106	0,26768819	TBX5;TBX5
cg11525285	0,538114767	0,272274974	0,26583979	VSX2
cg13028700	0,381839775	0,118693602	0,26314617	PAX6
cg08005992	0,431312117	0,171738709	0,25957341	PAX6;PAX6;PAX6
cg19290410	0,438220817	0,182859974	0,25536084	TBX5;TBX5
cg16559598	0,357787492	0,102540268	0,25524722	TBX5;TBX5
cg19787532	0,526737958	0,271689751	0,25504821	TBX5;TBX5;TBX5;TBX5
cg05769349	0,419101308	0,169629108	0,2494722	TBX5;TBX5
cg11176135	0,606070483	0,361079006	0,24499148	VSX2
cg17462200	0,524110725	0,279285183	0,24482554	TBX5;TBX5;TBX5;TBX5
cg21057587	0,550817558	0,307427329	0,24339023	VSX2
cg07660750	0,625609417	0,382805036	0,24280438	PAX6;PAX6;PAX6
cg26196480	0,58049075	0,340130849	0,2403599	TBX5;TBX5
cg12404281	0,867989375	0,628991383	0,23899799	RXRG;RXRG
cg19473653	0,808867592	0,570529551	0,23833804	RB1
cg12670347	0,627312883	0,38919388	0,238119	TBX5;TBX5
cg03786336	0,333961708	0,101258523	0,23270318	HES1
cg02642123	0,416624883	0,186803079	0,2298218	PAX6
cg12264949	0,343458058	0,114419919	0,22903814	PITX3
cg00756451	0,406566292	0,178822869	0,22774342	TBX5;TBX5;TBX5;TBX5
cg00509921	0,352680958	0,126193177	0,22648778	MAF;MAF;MAF
cg16330247	0,519119567	0,293422103	0,22569746	SOX11;SOX11
cg03843000	0,407967942	0,183269491	0,22469845	TBX5;TBX5
cg03126694	0,851426175	0,629771617	0,22165456	LPAR6;LPAR6;RB1
cg25598371	0,299713167	0,078113142	0,22160002	HES1;HES1
cg22045225	0,464358733	0,245617409	0,21874132	TBX5;TBX5;TBX5;TBX5

cg16458436	0,463011067	0,245049903	0,21796116	TBX5;TBX5
cg01904410	0,395917308	0,182200192	0,21371712	PAX6
cg03877376	0,410878333	0,198546749	0,21233158	TBX5;TBX5;TBX5;TBX5
cg09954698	0,684006117	0,472457866	0,21154825	VSX2
cg15778437	0,284155183	0,076129521	0,20802566	PAX6
cg23827572	0,372781264	0,168891968	0,2038893	TBX5;TBX5
cg08623787	0,41385335	0,210819794	0,20303356	RXRG;RXRG
cg21907579	0,394602208	0,193667246	0,20093496	TBX5;TBX5;TBX5;TBX5
cg20754348	0,564917183	0,364476463	0,20044072	NR2F2;NR2F2
cg25949958	0,2615278	0,061334066	0,20019373	PAX6;PAX6;PAX6
cg00642359	0,643842242	0,446633903	0,19720834	TBX5;TBX5;TBX5;TBX5
cg11717564	0,365612775	0,169669064	0,19594371	RXRG;RXRG
cg03578473	0,335549192	0,140802885	0,19474631	NEUROD1
cg16605327	0,348201417	0,157096753	0,19110466	TBX5;TBX5
cg00001874	0,769765433	0,582174974	0,18759046	ATOH7;ATOH7
cg14264795	0,797863783	0,611852954	0,18601083	TBX5;TBX5;TBX5
cg12324970	0,680399608	0,49597756	0,18442205	PITX3
cg11287400	0,843948408	0,661020423	0,18292799	MITF;MITF;MITF
cg23820885	0,414974908	0,234171214	0,18080369	TBX5;TBX5;TBX5;TBX5
cg08807097	0,304457817	0,133788987	0,17066883	TBX5;TBX5;TBX5
cg10281002	0,374594383	0,206861883	0,1677325	TBX5;TBX5
cg23669287	0,425230858	0,260743517	0,16448734	NR2F2;NR2F2
cg14002345	0,19439396	0,031620583	0,16277338	PAX6;PAX6;PAX6
cg11333459	0,30803005	0,151632709	0,15639734	PAX6
cg04685570	0,414513983	0,259211449	0,15530253	TBX5;TBX5;TBX5;TBX5
cg17826834	0,515959258	0,364144217	0,15181504	NR2F2
cg09042277	0,38430965	0,235494966	0,14881468	TBX5;TBX5;TBX5;TBX5
cg23097006	0,269967692	0,121371351	0,14859634	VSX1;VSX1

cg24143137	0,583961558	0,435982783	0,14797878	PITX3
cg11228052	0,313170183	0,166334853	0,14683533	ASCL1
cg17645823	0,4318797	0,286278657	0,14560104	TBX5;TBX5
cg03594550	0,727286117	0,585341391	0,14194473	SOX11;SOX11
cg22788908	0,765061833	0,625748929	0,1393129	POU2F1
cg06550984	0,311639442	0,173132934	0,13850651	PTF1A
cg06988349	0,665533158	0,528583954	0,1369492	ATOH7
cg06830500	0,514467258	0,378458846	0,13600841	SIX6
cg00182639	0,602841492	0,469356434	0,13348506	TBX5;TBX5;TBX5;TBX5
cg20709008	0,188613917	0,057585051	0,13102887	NEUROD1
cg19697981	0,271088233	0,142289007	0,12879923	NR2E1;NR2E1
cg27597956	0,269812392	0,141494553	0,12831784	PAX6;PAX6
cg20426571	0,225059308	0,096977433	0,12808188	TBX5;TBX5;TBX5
cg11162118	0,310020467	0,184101747	0,12591872	PAX6
cg25466368	0,205832025	0,082330831	0,12350119	HES1;HES1
cg04608900	0,3191676	0,197694045	0,12147355	VAX2
cg08391415	0,269147042	0,147972051	0,12117499	PAX6
cg06640206	0,854190842	0,733504454	0,12068639	MITF;MITF;MITF;MITF;MITF;MITF
cg05933765	0,802961883	0,68628672	0,11667516	ATOH7
cg16805360	0,833086833	0,721834286	0,11125255	TBX5;TBX5;TBX5
cg26659079	0,252261867	0,141098907	0,11116296	SOX11;SOX11
cg15846787	0,299417458	0,191505431	0,10791203	VAX2
cg04829946	0,218658692	0,115089882	0,10356881	PTF1A
cg06559274	0,165016592	0,063455981	0,10156061	NR2E1;NR2E1
cg26315277	0,241783083	0,141219841	0,10056324	PAX6
cg14443953	0,301234108	0,201160046	0,10007406	PAX6
cg04580872	0,268168433	0,169559946	0,09860849	HSF4;HSF4
cg08383063	0,173219083	0,074768636	0,09845045	RB1

cg02278499	0,201852533	0,103458031	0,0983945	NR2F2;NR2F2;NR2F2;NR2F2;MIR1469;NR2F2
cg18897632	0,161230383	0,063056031	0,09817435	SOX11;SOX11
cg03585237	0,875051592	0,778072086	0,09697951	RXRG;RXRG
cg00806138	0,796090875	0,699403951	0,09668692	FOXE3
cg09795588	0,168208908	0,074296971	0,09391194	PTF1A
cg13265869	0,324015275	0,233244082	0,09077119	VAX2
cg11038507	0,857580825	0,766939246	0,09064158	MITF;MITF;MITF;MITF;MITF;MITF
cg00333843	0,778879883	0,689363566	0,08951632	MAF;MAF;MAF
cg13964092	0,176960017	0,087577934	0,08938208	LHX2
cg20131194	0,138114878	0,051268245	0,08684663	PAX6;PAX6;PAX6
cg08526991	0,30611535	0,22043982	0,08567553	SOX11
cg26334672	0,384511592	0,299284149	0,08522744	VAX2
cg27256523	0,1618692	0,077897013	0,08397219	RB1
cg14431443	0,486997342	0,40338716	0,08361018	TBX5;TBX5
cg00078318	0,176176425	0,092699857	0,08347657	SOX11;SOX11
cg19425332	0,317910808	0,234754077	0,08315673	VAX1;VAX1
cg12374431	0,279739883	0,198861772	0,08087811	VAX2
cg20320283	0,898163625	0,817663109	0,08050052	FOXE3
cg20156072	0,788254667	0,707904397	0,08035027	ATOH7
cg09123368	0,245987342	0,167174621	0,07881272	VAX1;VAX1
cg13743393	0,125828803	0,04739828	0,07843052	RB1
cg25032342	0,412010942	0,333714251	0,07829669	VAX2
cg19161112	0,170768555	0,093850683	0,07691787	PTF1A
cg09232021	0,841158992	0,764974106	0,07618489	MAF;MAF;MAF
cg12961656	0,22370415	0,149319841	0,07438431	HES1
cg26593946	0,430019842	0,355657037	0,0743628	NR2F2
cg16822387	0,233994625	0,160288247	0,07370638	PAX6;PAX6;PAX6
cg18058532	0,143702819	0,070296849	0,07340597	PAX6;PAX6;PAX6;PAX6

cg14205663	0,580756908	0,507488723	0,07326819	NR2F2;NR2F2
cg23320862	0,20560295	0,133158531	0,07244442	TBX5;TBX5;TBX5
cg22827124	0,287342292	0,217019443	0,07032285	VAX2
cg16113298	0,147265579	0,077382302	0,06988328	PAX6;PAX6;PAX6;PAX6
cg15034345	0,117129191	0,047896605	0,06923259	SOX11;SOX11
cg06834240	0,232017025	0,164387733	0,06762929	MAF;MAF;MAF
cg03527353	0,15439233	0,087914869	0,06647746	PAX6;PAX6;PAX6;PAX6
cg02836919	0,166919198	0,100589931	0,06632927	PTF1A
cg15379412	0,245330908	0,179552074	0,06577883	NR2F2;NR2F2;NR2F2;NR2F2;MIR1469;NR2F2
cg03018496	0,487677083	0,421945894	0,06573119	NR2F2
cg04308089	0,442145075	0,377078829	0,06506625	NR2F2
cg12198198	0,844039467	0,78043678	0,06360269	MITF;MITF;MITF;MITF
cg01863682	0,176261696	0,112797854	0,06346384	NEUROD1
cg09969043	0,38570245	0,322333317	0,06336913	NR2F2
cg19456540	0,117753757	0,054476472	0,06327728	SIX6
cg13901501	0,171684688	0,108577272	0,06310742	RXRG;RXRG
cg07747635	0,140868632	0,078013023	0,06285561	HES1
cg14776201	0,155211602	0,093956997	0,06125461	SOX11;SOX11
cg02679809	0,128122783	0,068660885	0,0594619	PAX6
cg01637131	0,145772794	0,087092767	0,05868003	HES1
cg16905280	0,82707915	0,770998791	0,05608036	MITF;MITF;MITF;MITF;MITF;MITF
cg05091519	0,090095702	0,034543069	0,05555263	PAX6
cg25540028	0,856450283	0,801179689	0,05527059	LPAR6;LPAR6;RB1
cg22882178	0,142838372	0,088795888	0,05404248	PITX3;PITX3
cg16985320	0,107385505	0,053360735	0,05402477	RB1
cg26212180	0,212644866	0,158640135	0,05400473	VAX1;VAX1
cg04626565	0,144672069	0,093368377	0,05130369	MAF;MAF
cg11973244	0,198554217	0,147327387	0,05122683	PAX6;PAX6;PAX6

cg10700564	0,170879937	0,120055426	0,05082451	ASCL1
cg20487710	0,189999197	0,140370833	0,04962836	PTF1A
cg04132983	0,262341125	0,21295956	0,04938157	MAF;MAF;MAF
cg00367497	0,143813826	0,095308799	0,04850503	NR2F2;NR2F2;NR2F2;MIR1469;NR2F2
cg00060304	0,184737043	0,136859316	0,04787773	PAX6
cg11716272	0,174105343	0,126247207	0,04785814	SOX11;SOX11
cg14414792	0,617717125	0,571370666	0,04634646	ATOH7
cg06173889	0,5194547	0,475566223	0,04388848	SOX11;SOX11
cg07456585	0,7154385	0,671630997	0,0438075	VSX2
cg08644711	0,089680978	0,045941843	0,04373913	RB1
cg12156885	0,351501492	0,309012846	0,04248865	LHX1;LHX1
cg01913568	0,092156872	0,049780583	0,04237629	RXRG;RXRG
cg03811260	0,136117653	0,09521872	0,04089893	HSF4;HSF4
cg11828108	0,150393637	0,109843271	0,04055037	PITX3
cg18614734	0,149713593	0,10973349	0,0399801	NR2F2;NR2F2;NR2F2;MIR1469;NR2F2
cg00130817	0,893993958	0,854387586	0,03960637	MITF;MITF;MITF;MITF
cg20053158	0,164436313	0,124882449	0,03955386	ASCL1
cg14037837	0,186484118	0,147565621	0,0389185	PAX6
cg17015844	0,2465923	0,20794121	0,03865109	ASCL1
cg23933618	0,174276427	0,137719184	0,03655724	PAX6
cg15449049	0,779041345	0,742826474	0,03621487	MITF;MITF
cg05945059	0,126062965	0,090400191	0,03566277	RAX;RAX
cg19958021	0,826707783	0,791599163	0,03510862	SOX11;SOX11
cg09822018	0,114234516	0,079665797	0,03456872	MAF;MAF
cg05108467	0,350473058	0,316649826	0,03382323	NR2F2
cg23763043	0,089111843	0,05539924	0,0337126	NR2F2;NR2F2;NR2F2;MIR1469;NR2F2
cg07504616	0,118326327	0,084797388	0,03352894	MAF;MAF
cg05671070	0,195982008	0,162945958	0,03303605	FLJ45983;GATA3;GATA3;FLJ45983

cg17514495	0,894327117	0,861672429	0,03265469	RXRG;RXRG
cg00268831	0,78712955	0,755196906	0,03193264	LPAR6;LPAR6;RB1
cg07905944	0,126901167	0,094994942	0,03190622	PAX6
cg13065537	0,119227886	0,088478738	0,03074915	HES1
cg07091781	0,873100375	0,842678746	0,03042163	RXRG;RXRG
cg27649073	0,153270675	0,123131813	0,03013886	PITX3
cg10702309	0,21576036	0,186070565	0,0296898	HES1
cg14055374	0,078150005	0,048542096	0,02960791	MAF;MAF;MAF;MAF
cg03111498	0,142103125	0,112791023	0,0293121	VSX1;VSX1
cg18716076	0,264500025	0,235214157	0,02928587	ISL1
cg13325154	0,074651699	0,045639366	0,02901233	PITX3
cg05790989	0,7629622	0,733977854	0,02898435	POU2F1
cg06621126	0,120913269	0,09196385	0,02894942	HSF4;HSF4
cg16655390	0,818397142	0,789573566	0,02882358	MITF;MITF;MITF;MITF
cg06230736	0,179807019	0,151274083	0,02853294	FLJ45983;GATA3;GATA3;FLJ45983
cg20278291	0,165090067	0,13694675	0,02814332	OTX2;OTX2;OTX2
cg19244064	0,86190505	0,83409712	0,02780793	PITX3
cg02162324	0,515523633	0,488256363	0,02726727	HSF4;FBXL8;HSF4
cg22675486	0,113985406	0,086773476	0,02721193	RXRG;RXRG
cg10689889	0,108275316	0,081385563	0,02688975	MAF;MAF
cg15909737	0,88163705	0,855050712	0,02658634	MITF;MITF
cg18639233	0,072447619	0,046286944	0,02616068	SIX6
cg04255278	0,17030782	0,144906105	0,02540172	FLJ42709;FLJ42709;NR2F1
cg14681055	0,115552745	0,091021049	0,0245317	PITX3
cg00873937	0,116650557	0,092295223	0,02435533	ATOH7;ATOH7
cg21764190	0,140007166	0,115995566	0,0240116	PAX6
cg02962380	0,088279094	0,064643163	0,02363593	MAF;MAF
cg16448525	0,123158575	0,099710751	0,02344782	FLJ42709;FLJ42709;NR2F1

cg10177238	0,113142786	0,089952945	0,02318984	PAX6;PAX6;PAX6
cg00397673	0,223928233	0,200959766	0,02296847	RAX
cg18187680	0,189661458	0,167366207	0,02229525	FLJ45983;GATA3;GATA3;FLJ45983
cg17885806	0,118880128	0,097549397	0,02133073	RXRG;RXRG
cg15442419	0,125327506	0,104393799	0,02093371	TFAP2A;TFAP2A;TFAP2A
cg01559904	0,752684133	0,732334263	0,02034987	LPAR6;LPAR6;RB1
cg01049775	0,215896358	0,195627831	0,02026853	ATF4;ATF4
cg11575738	0,132507436	0,112522161	0,01998527	DLX1;DLX1
cg21428647	0,133303708	0,113737973	0,01956573	HES1
cg16556111	0,128805285	0,109273745	0,01953154	NR2F1
cg14611174	0,128560468	0,109423671	0,0191368	SIX6
cg18904346	0,145426052	0,126362068	0,01906398	LHX2
cg23706497	0,135231333	0,116330057	0,01890128	OTX2;OTX2;OTX2
cg03492327	0,294583033	0,27594254	0,01864049	OTX2;OTX2
cg05945291	0,128457184	0,110453707	0,01800348	NR2F1
cg08097882	0,138065173	0,120544383	0,01752079	POU4F1;POU4F1
cg05277991	0,101481398	0,083984337	0,01749706	MITF
cg26896762	0,16691736	0,149533538	0,01738382	ISL1;ISL1
cg23374964	0,169214678	0,1520289	0,01718578	VAX1;VAX1
cg23087020	0,182494259	0,165609657	0,0168846	POU2F1
cg01865113	0,084647824	0,067860076	0,01678775	SOX9
cg04736351	0,232306981	0,215767834	0,01653915	OTX2
cg16315058	0,122184748	0,106250008	0,01593474	TBX5;TBX5;TBX5;TBX5
cg09728012	0,073620971	0,058227723	0,01539325	FLJ45983;GATA3;GATA3;FLJ45983
cg02788090	0,135812872	0,120548085	0,01526479	SOX9
cg23042706	0,1243642	0,109110716	0,01525348	NR2F2;NR2F2;NR2F2;MIR1469;NR2F2
cg07231544	0,086467071	0,071314535	0,01515254	MITF
cg08475576	0,090769546	0,075724806	0,01504474	MAF;MAF

cg11731114	0,095807538	0,080870674	0,01493686	FLJ45983;GATA3;GATA3;FLJ45983
cg08778908	0,622275908	0,607393531	0,01488238	LPAR6;LPAR6;LPAR6;RB1
cg21503826	0,070890853	0,05639219	0,01449866	HES1;HES1
cg15070718	0,047965212	0,033661831	0,01430338	NR2F2;NR2F2;NR2F2;NR2F2;MIR1469
cg27294837	0,208887668	0,194738279	0,01414939	POU4F1
cg06652112	0,038537366	0,024429336	0,01410803	HES1
cg02978827	0,094520735	0,080632882	0,01388785	RB1
cg20528093	0,324467783	0,310799974	0,01366781	PAX6
cg03383382	0,148672418	0,135188235	0,01348418	NR2E1;NR2E1
cg06414605	0,069265517	0,055794719	0,0134708	HES1
cg19959647	0,081645894	0,068363027	0,01328287	HES1
cg04847548	0,07137048	0,05809041	0,01328007	FLJ45983;GATA3;GATA3;FLJ45983
cg17389519	0,24949515	0,236461937	0,01303321	PTF1A
cg07548367	0,152887516	0,140215544	0,01267197	DLX5
cg06881580	0,091078675	0,078932987	0,01214569	MAF;MAF
cg10040836	0,127409317	0,115579288	0,01183003	NR2F2;NR2F2;NR2F2;MIR1469;NR2F2
cg14214262	0,050894125	0,039084204	0,01180992	TBX5;TBX5;TBX5;TBX5
cg08553999	0,070244499	0,058685955	0,01155854	MAF;MAF
cg03719083	0,1449045	0,133349406	0,01155509	TFAP2A;TFAP2A;TFAP2A
cg01224891	0,055611852	0,044279805	0,01133205	FLJ45983;GATA3;GATA3;FLJ45983
cg20875159	0,127818469	0,116885782	0,01093269	TBX5;TBX5;TBX5;TBX5
cg04050331	0,071810309	0,061154024	0,01065628	FLJ45983;GATA3;GATA3;FLJ45983
cg27019126	0,077105198	0,066454552	0,01065065	RAX
cg02376553	0,171154717	0,160598917	0,0105558	TFAP2A;TFAP2A;TFAP2A
cg07846755	0,130925458	0,12050023	0,01042523	HES1
cg11451367	0,091194462	0,081047557	0,01014691	NR2F1
cg25962286	0,066658242	0,056565531	0,01009271	DLX2
cg07242563	0,047688482	0,037775875	0,00991261	MAF;MAF

cg07480495	0,078056778	0,068217832	0,00983895	FOXN4
cg05321656	0,050017041	0,040251479	0,00976556	ATOH7;ATOH7
cg20518889	0,11563317	0,105978648	0,00965452	VAX2
cg06018531	0,09304946	0,083427634	0,00962183	MAF;MAF;MAF;MAF
cg05127369	0,090210905	0,080598743	0,00961216	NR2F2;NR2F2;NR2F2;NR2F2;MIR1469
cg03847535	0,863289617	0,853969969	0,00931965	MITF;MITF;MITF
cg13361843	0,151578692	0,142396597	0,00918209	VAX2
cg02397497	0,127761327	0,118616804	0,00914452	TFAP2A;TFAP2A;TFAP2A;TFAP2A
cg23484599	0,690712308	0,681716449	0,00899586	PAX6;PAX6;PAX6
cg09252999	0,1326199	0,123731476	0,00888842	PAX6
cg13017614	0,682721975	0,673840434	0,00888154	FOXE3
cg09500815	0,063148906	0,05430391	0,008845	NR2F2;NR2F2;NR2F2;NR2F2;MIR1469
cg16048568	0,053617017	0,044936561	0,00868046	NR2F2;NR2F2;NR2F2;NR2F2;MIR1469;NR2F2
cg21038382	0,070334718	0,061869771	0,00846495	DLX1;DLX1;DLX1;DLX1
cg08724563	0,077432724	0,069112988	0,00831974	RAX
cg10581281	0,101971852	0,093709731	0,00826212	MITF
cg20189674	0,235436517	0,227368409	0,00806811	LHX1;LHX1
cg13685139	0,80342815	0,795471851	0,0079563	MITF;MITF
cg16329784	0,053572965	0,045854244	0,00771872	PITX3
cg12795952	0,059452711	0,052296596	0,00715612	HES1
cg20314737	0,116652656	0,109570005	0,00708265	FLJ45983;GATA3;GATA3;FLJ45983
cg04249993	0,817928083	0,811041026	0,00688706	LPAR6;RB1
cg13431023	0,108896977	0,102083725	0,00681325	FLJ45983;GATA3;GATA3;FLJ45983
cg18710462	0,099837717	0,093057661	0,00678006	NR2E1
cg20784263	0,056494591	0,0497592	0,00673539	RB1
cg13264800	0,832672117	0,825942974	0,00672914	MITF;MITF;MITF;MITF
cg23058185	0,114706611	0,107982657	0,00672395	FLJ45983;GATA3;GATA3;FLJ45983
cg05840031	0,115169948	0,108667099	0,00650285	PAX6;PAX6;PAX6

cg04982951	0,1875345	0,181128197	0,0064063	FLJ45983;GATA3;GATA3;FLJ45983
cg06327596	0,091873172	0,085506268	0,0063669	MAF;MAF;MAF;MAF
cg15330117	0,128285048	0,121972095	0,00631295	FLJ45983;GATA3;GATA3;GATA3;GATA3;FLJ45983
cg05505872	0,102514799	0,096217274	0,00629753	NR2F2;NR2F2
cg24528447	0,101900879	0,095797375	0,0061035	NR2F1
cg07094440	0,339559742	0,33365022	0,00590952	VAX2
cg05164666	0,160592302	0,154737641	0,00585466	VAX2
cg00822495	0,124491882	0,118702234	0,00578965	OTX2
cg06070625	0,704237225	0,698462814	0,00577441	MITF;MITF
cg18554811	0,057658763	0,052010503	0,00564826	RB1
cg19894747	0,042139778	0,036505371	0,00563441	FLJ45983;GATA3;GATA3;FLJ45983
cg04002444	0,133067571	0,127653155	0,00541442	TBX5;TBX5;TBX5;TBX5
cg16921310	0,048462182	0,043054361	0,00540782	ASCL1;ASCL1
cg14719076	0,085945737	0,080721496	0,00522424	DLX5
cg04043795	0,04235767	0,037294788	0,00506288	SOX11;SOX11
cg04858597	0,042918517	0,037869429	0,00504909	MAF;MAF
cg26848086	0,070280636	0,065434379	0,00484626	PAX6;PAX6;PAX6
cg15871749	0,040425809	0,03564527	0,00478054	HES1
cg24647276	0,071847421	0,067090975	0,00475645	FLJ45983;GATA3;GATA3;FLJ45983
cg00096922	0,117633674	0,112896295	0,00473738	DLX5
cg10356613	0,046151891	0,04161818	0,00453371	LHX1
cg12377424	0,063195198	0,058665077	0,00453012	TFAP2A;TFAP2A;TFAP2A;TFAP2A
cg16712637	0,040570598	0,036060718	0,00450988	ASCL1;ASCL1
cg08763161	0,098936288	0,094433859	0,00450243	TFAP2A;TFAP2A;TFAP2A
cg12730771	0,091503618	0,087031224	0,00447239	FLJ45983;GATA3;GATA3;FLJ45983
cg20576153	0,148535773	0,144146261	0,00438951	SIX6
cg22766818	0,032969882	0,028820952	0,00414893	RB1
cg08128228	0,074069728	0,069952424	0,0041173	POU2F1

cg11078683	0,057759875	0,053743053	0,00401682	LHX1
cg19600750	0,078357218	0,074421115	0,0039361	OTX2
cg10552385	0,025323277	0,021400012	0,00392326	RB1;RB1
cg25734490	0,03570183	0,032021409	0,00368042	ASCL1
cg13535663	0,05965518	0,055981949	0,00367323	POU2F1
cg25940847	0,121424426	0,117956636	0,00346779	TFAP2A;TFAP2A;TFAP2A;TFAP2A
cg02113449	0,083908158	0,080505794	0,00340236	RB1
cg09288780	0,112673225	0,109414614	0,00325861	TFAP2A;TFAP2A;TFAP2A
cg02152992	0,091818298	0,088720628	0,00309767	RB1
cg22977876	0,051310318	0,048219005	0,00309131	DLX1;DLX1
cg00577588	0,063949178	0,060874375	0,0030748	ATF4;ATF4
cg09793118	0,183918675	0,18086594	0,00305274	PITX3
cg12908952	0,082111343	0,079171209	0,00294013	POU2F1
cg23435080	0,092271883	0,089396769	0,00287511	HES1
cg03595100	0,134242408	0,131368098	0,00287431	VAX2
cg19832172	0,065421933	0,06254861	0,00287332	ATF4;ATF4
cg25977304	0,041530132	0,03885638	0,00267375	POU2F1
cg12491991	0,033448494	0,03082075	0,00262774	ATF4;ATF4
cg09150117	0,050780198	0,048199794	0,0025804	DLX5
cg02250263	0,070815375	0,068326277	0,0024891	SOX9
cg01370181	0,028215246	0,025756442	0,0024588	NR2F2;NR2F2
cg00801465	0,109643863	0,107237704	0,00240616	HES1
cg05527869	0,048285388	0,046017272	0,00226812	LHX1
cg23273935	0,100270437	0,098011649	0,00225879	TFAP2A;TFAP2A;TFAP2A;TFAP2A
cg24182293	0,059839913	0,057613232	0,00222668	HES1
cg10043865	0,041962001	0,039814851	0,00214715	LHX1
cg26691604	0,066362823	0,064263267	0,00209956	MEIS1
cg21182322	0,070537838	0,068539768	0,00199807	POU2F1

cg04993975	0,041168923	0,039198735	0,00197019	LHX1
cg03700449	0,067224242	0,06529782	0,00192642	ASCL1
cg17423711	0,088187586	0,086330451	0,00185714	POU2F1;POU2F1
cg07320819	0,028471676	0,026635196	0,00183648	RB1;RB1
cg08132837	0,102758679	0,100936838	0,00182184	SIX6
cg06472439	0,150170154	0,14836095	0,0018092	NR2F1
cg15852223	0,026656973	0,024859159	0,00179781	FLJ45983;GATA3;GATA3;FLJ45983
cg18555811	0,041510518	0,039712963	0,00179755	HES1
cg13447454	0,870450167	0,868664063	0,0017861	POU2F1
cg24425021	0,035086371	0,033394985	0,00169139	POU4F1;POU4F1
cg04122974	0,031048248	0,029404675	0,00164357	ATF4;ATF4
cg10638605	0,043669074	0,042085555	0,00158352	ATOH7
cg27569040	0,07378554	0,072202265	0,00158328	ASCL1;ASCL1
cg00838150	0,094488299	0,09290901	0,00157929	MITF
cg07270078	0,049036527	0,047467368	0,00156916	ISL1
cg06259996	0,082671248	0,081142233	0,00152901	NR2F1
cg22326903	0,040903598	0,039405657	0,00149794	FOXN4;FOXN4
cg12439899	0,109470391	0,107986801	0,00148359	TFAP2A;TFAP2A;TFAP2A
cg24937706	0,06587028	0,064473293	0,00139699	RB1
cg06933899	0,064021024	0,062697313	0,00132371	ATF4;ATF4;ATF4
cg24761507	0,047050853	0,045758415	0,00129244	LHX1
cg07026599	0,049471258	0,048302703	0,00116855	MAF;MAF
cg26866482	0,048406948	0,047289706	0,00111724	ASCL1
cg15187550	0,027468952	0,026471495	0,00099746	FLJ45983;GATA3;GATA3;FLJ45983
cg05356738	0,037781907	0,036832751	0,00094916	FLJ45983;GATA3;GATA3;FLJ45983
cg01001468	0,028092807	0,027312835	0,00077997	MAF;MAF
cg09246833	0,127115693	0,126343392	0,0007723	ATF4;ATF4
cg03141620	0,041649519	0,040911649	0,00073787	DLX1;DLX1

cg23763424	0,039237325	0,038524663	0,00071266	DLX2
cg01366246	0,067617851	0,067025703	0,00059215	HES1
cg02968429	0,027218462	0,026729909	0,00048855	NR2F2;NR2F2
cg01369278	0,046349404	0,045957955	0,00039145	DLX2
cg17566118	0,05951202	0,059301921	0,0002101	FLJ45983;GATA3;GATA3;FLJ45983
cg23644992	0,332056133	0,33189838	0,00015775	VAX2
cg27420520	0,026192201	0,02606847	0,00012373	ASCL1
cg08707112	0,115844144	0,115833409	1,0735E-05	FLJ45983;GATA3;GATA3;FLJ45983
cg08581737	0,069276685	0,069285051	-8,3661E-06	DLX2
cg10042106	0,062692563	0,062713189	-2,0627E-05	OTX2
cg20699736	0,152962673	0,153023502	-6,083E-05	VAX2
cg07516470	0,036307481	0,036415918	-0,00010844	FLJ45983;GATA3;GATA3;FLJ45983
cg02902395	0,025551539	0,025695191	-0,00014365	POU2F1
cg11826116	0,079321068	0,079464922	-0,00014385	ATF4;ATF4
cg01994205	0,116706779	0,116857237	-0,00015046	POU4F1;POU4F1
cg02246645	0,076083595	0,076386282	-0,00030269	ASCL1
cg13252294	0,036674226	0,037015386	-0,00034116	ATF4;ATF4
cg05071898	0,033101172	0,033500731	-0,00039956	FLJ45983;GATA3;GATA3;FLJ45983
cg19679989	0,056560228	0,057010414	-0,00045019	FLJ45983;GATA3;GATA3;FLJ45983
cg01715455	0,083760379	0,084249749	-0,00048937	VSX1;VSX1
cg27331225	0,038482994	0,03911699	-0,000634	HES1
cg16995983	0,049192168	0,049861736	-0,00066957	NR2F1;NR2F1
cg22154024	0,022483333	0,02315876	-0,00067543	ASCL1
cg00349776	0,101460071	0,102148745	-0,00068867	RXRG;RXRG;RXRG;RXRG
cg26937148	0,088080516	0,088848801	-0,00076829	NR2F1;NR2F1
cg04126852	0,067279348	0,06814949	-0,00087014	POU2F1
cg07747970	0,175597627	0,17647066	-0,00087303	LHX1
cg19315863	0,02710493	0,02807576	-0,00097083	FLJ45983;GATA3;GATA3;FLJ45983

cg14009588	0,082596655	0,08357354	-0,00097688	TFAP2A;TFAP2A
cg15268622	0,042670002	0,043685821	-0,00101582	POU2F1
cg18619591	0,062435292	0,063533355	-0,00109806	DLX1;DLX1
cg14641315	0,034563813	0,035705159	-0,00114135	RB1
cg08721076	0,023967888	0,025121413	-0,00115353	DLX2
cg25444231	0,022378812	0,023542568	-0,00116376	HES1
cg15326320	0,018921441	0,020274458	-0,00135302	DLX2
cg07578663	0,036507783	0,037961828	-0,00145404	FLJ45983;GATA3;GATA3;FLJ45983
cg00296158	0,149662387	0,151124184	-0,0014618	POU2F1
cg13485756	0,100114119	0,101583034	-0,00146892	RB1
cg02622074	0,071920079	0,073523051	-0,00160297	TFAP2A;TFAP2A;TFAP2A;TFAP2A
cg21814667	0,07572546	0,077340386	-0,00161493	RB1
cg13974768	0,065761404	0,067380365	-0,00161896	POU2F1
cg27144395	0,083074344	0,084943023	-0,00186868	FOXN4
cg13462160	0,373035908	0,374923423	-0,00188751	ATF4;ATF4;ATF4;ATF4
cg24799863	0,174970251	0,176906203	-0,00193595	DLX5;DLX5
cg06996609	0,091914261	0,093870749	-0,00195649	DLX1;DLX1
cg12312988	0,081322656	0,083345325	-0,00202267	SOX11
cg01111397	0,018967384	0,021013497	-0,00204611	DLX2
cg22356339	0,045035194	0,047127064	-0,00209187	ASCL1
cg13344740	0,127880058	0,130040154	-0,0021601	DLX5;DLX5
cg18544329	0,150051654	0,152289981	-0,00223833	DLX5
cg12279877	0,055907979	0,058251575	-0,0023436	LHX1
cg20718350	0,032468273	0,034838635	-0,00237036	ASCL1
cg07885289	0,039933882	0,042322148	-0,00238827	LHX1;LHX1
cg25472998	0,030056999	0,032466114	-0,00240911	TFAP2A;TFAP2A;TFAP2A
cg18738647	0,125408141	0,127951088	-0,00254295	FLJ45983;GATA3;GATA3;FLJ45983
cg23943136	0,052390274	0,055008167	-0,00261789	FLJ45983;GATA3;GATA3;FLJ45983

cg12463600	0,085626981	0,088278802	-0,00265182	NR2E1
cg03074888	0,084803355	0,087475981	-0,00267263	RAX
cg06963053	0,029643841	0,032353353	-0,00270951	NEUROD1
cg14763548	0,035357719	0,038097839	-0,00274012	VSX1;VSX1
cg16663344	0,115271331	0,118050199	-0,00277887	NR2F1;NR2F1
cg15579817	0,070544166	0,073454268	-0,0029101	VAX2
cg25662463	0,041460863	0,044463168	-0,00300231	VAX1;VAX1
cg21169969	0,112816841	0,115833026	-0,00301619	FOXN4
cg20361600	0,034075308	0,037123398	-0,00304809	DLX2
cg03048535	0,084159768	0,087224599	-0,00306483	FOXN4
cg01166071	0,058168554	0,061263361	-0,00309481	FLJ45983;GATA3;GATA3;FLJ45983
cg22836400	0,067654078	0,070854027	-0,00319995	TFAP2A;TFAP2A
cg01778450	0,060229365	0,063466772	-0,00323741	NR2F1;NR2F1
cg18270629	0,024063105	0,027383316	-0,00332021	PAX6;PAX6;PAX6
cg18920423	0,021311685	0,024708113	-0,00339643	PTF1A
cg14794428	0,083690323	0,08724814	-0,00355782	ASCL1
cg05930133	0,051902885	0,055472154	-0,00356927	MEIS1
cg03340014	0,21133015	0,214992823	-0,00366267	CRX;CRX
cg15457058	0,021628443	0,025407916	-0,00377947	FOXE3
cg20691283	0,080076878	0,083875794	-0,00379892	MEIS1
cg10008757	0,146039341	0,149866889	-0,00382755	GATA3;GATA3
cg08347183	0,07530305	0,07915232	-0,00384927	FLJ45983;GATA3;GATA3;FLJ45983
cg18759960	0,101965268	0,106108835	-0,00414357	PTF1A
cg06785999	0,036385517	0,040554811	-0,00416929	SIX6;SIX6
cg11438428	0,164371123	0,168706941	-0,00433582	PTF1A
cg20014049	0,023203363	0,02755903	-0,00435567	PTF1A
cg08905496	0,121005322	0,125400427	-0,0043951	RXRG;RXRG;RXRG;RXRG
cg14981137	0,097579853	0,10200503	-0,00442518	ISL1

cg06424860	0,081522388	0,086046713	-0,00452432	ISL1
cg17460737	0,083327908	0,08803479	-0,00470688	HES1
cg01779747	0,080401423	0,085171091	-0,00476967	FOXN4
cg08327269	0,030004153	0,034808259	-0,00480411	NEUROD1
cg06676637	0,149562997	0,154637803	-0,00507481	RAX
cg09157727	0,075072563	0,080186462	-0,0051139	NR2F2;NR2F2
cg06830064	0,881268067	0,886539849	-0,00527178	SOX11;SOX11
cg13457172	0,038753361	0,044167263	-0,0054139	VAX1;VAX1
cg02609473	0,101656861	0,107124935	-0,00546807	MEIS1
cg24767148	0,041974159	0,04749883	-0,00552467	PTF1A
cg08530978	0,059574084	0,065220815	-0,00564673	DLX1;DLX1
cg11580493	0,059721152	0,065449195	-0,00572804	NR2F2;NR2F2
cg16652259	0,164767711	0,170613842	-0,00584613	DLX1;DLX1
cg05490712	0,07415719	0,080311957	-0,00615477	PAX6
cg03053826	0,214749225	0,22091274	-0,00616352	NR2F2
cg16249035	0,08719032	0,09339131	-0,00620099	TBX5;TBX5;TBX5;TBX5
cg21637670	0,0388449	0,045072061	-0,00622716	VAX2
cg11997899	0,110378238	0,116800736	-0,0064225	DLX5
cg09942248	0,033647142	0,040166377	-0,00651924	NEUROD1
cg07870982	0,047784224	0,054308937	-0,00652471	MAF;MAF
cg04030401	0,414697283	0,421288346	-0,00659106	HSF4;FBXL8;HSF4
cg12207450	0,115025838	0,121628326	-0,00660249	NR2F1;NR2F1
cg25079102	0,032111366	0,038746034	-0,00663467	TBX5;TBX5;TBX5;TBX5
cg18918349	0,055230479	0,061966192	-0,00673571	NR2E1
cg19809499	0,028316481	0,035123534	-0,00680705	FOXE3
cg21284680	0,063516456	0,070365876	-0,00684942	TFAP2A;TFAP2A;TFAP2A;TFAP2A
cg18093751	0,099056273	0,106026511	-0,00697024	NR2E1
cg09010671	0,031741394	0,038728224	-0,00698683	POU4F1

cg16756631	0,091269832	0,098298221	-0,00702839	DLX2;DLX2
cg13694576	0,139931135	0,147019226	-0,00708809	PAX6
cg24104938	0,127800799	0,135040086	-0,00723929	NR2E1
cg17891011	0,124012268	0,131600804	-0,00758854	FLJ45983;GATA3;GATA3;FLJ45983
cg27398263	0,031407433	0,039114387	-0,00770695	POU4F1
cg06926934	0,028326986	0,036035542	-0,00770856	NR2F2;NR2F2;NR2F2;MIR1469;NR2F2
cg03086663	0,139051166	0,146777563	-0,0077264	NR2F1
cg12975220	0,04553793	0,053400101	-0,00786217	MEIS1;MEIS1
cg13437635	0,876236192	0,884109463	-0,00787327	PITX3
cg14858469	0,078670675	0,086735992	-0,00806532	NR2F2;NR2F2
cg26292521	0,041859228	0,050127738	-0,00826851	FLJ45983;GATA3;GATA3;FLJ45983
cg13784080	0,08941593	0,097908449	-0,00849252	OTX2
cg10364513	0,064897936	0,073464551	-0,00856662	RXRG;RXRG;RXRG;RXRG
cg18400845	0,101528928	0,110099661	-0,00857073	PAX6
cg18983672	0,868504275	0,877110646	-0,00860637	FOXE3
cg15604467	0,099063177	0,107670766	-0,00860759	POU4F1
cg17082803	0,04866685	0,057397421	-0,00873057	VAX2
cg23885415	0,068178652	0,077048896	-0,00887024	VAX2
cg01153166	0,088897553	0,097885243	-0,00898769	NR2F2;NR2F2;NR2F2;NR2F2
cg14773685	0,07282265	0,081827648	-0,009005	HES1
cg16871519	0,0533255	0,062415818	-0,00909032	TFAP2A;TFAP2A
cg22647713	0,054528868	0,063854337	-0,00932547	FLJ45983;GATA3;GATA3;FLJ45983
cg17856083	0,88122445	0,890552651	-0,0093282	POU2F1
cg00286388	0,86275835	0,872173123	-0,00941477	LPAR6;RB1
cg16416045	0,03921522	0,048752661	-0,00953744	DLX2;DLX2
cg05098471	0,055991857	0,065637597	-0,00964574	MEIS1
cg13814485	0,043914479	0,053963239	-0,01004876	FLJ45983;GATA3;GATA3;FLJ45983
cg04943986	0,064327951	0,074447687	-0,01011974	NR2F2;NR2F2

cg11039614	0,024616332	0,034862162	-0,01024583	ISL1;ISL1
cg08927739	0,142245858	0,152694476	-0,01044862	ASCL1
cg14246617	0,057128343	0,067588442	-0,0104601	MITF
cg24225517	0,087106364	0,097621883	-0,01051552	NR2E1
cg18599069	0,137083585	0,147782115	-0,01069853	GATA3;GATA3
cg17957186	0,075079863	0,085779948	-0,01070008	POU4F1
cg27122213	0,072364886	0,083073288	-0,0107084	OTX2
cg02062480	0,153334792	0,164047429	-0,01071264	NR2E1;NR2E1
cg07300178	0,047021652	0,057843867	-0,01082222	VAX2
cg21264189	0,064863561	0,075753311	-0,01088975	POU4F1
cg17205316	0,071601071	0,082499094	-0,01089802	OTX2
cg21512370	0,56423075	0,575242637	-0,01101189	LPAR6;LPAR6;RB1
cg26339504	0,090795518	0,101899317	-0,0111038	VAX1;VAX1
cg27378835	0,106503693	0,117652678	-0,01114898	DLX1;DLX1
cg11904056	0,120375735	0,131587932	-0,0112122	NR2F1
cg01158822	0,082557534	0,09387889	-0,01132136	TFAP2A;TFAP2A
cg10755973	0,022578915	0,034164496	-0,01158558	LHX2
cg00043788	0,126883795	0,138561696	-0,0116779	VSX1;VSX1;VSX1;VSX1
cg17780624	0,080108138	0,091955403	-0,01184727	DLX1;DLX1
cg17576288	0,041786388	0,053643339	-0,01185695	POU4F1
cg06042480	0,153329708	0,165306116	-0,01197641	LHX1
cg03707948	0,102690487	0,11495517	-0,01226468	DLX2
cg08985029	0,034449134	0,04683299	-0,01238386	TBX5;TBX5;TBX5
cg20401252	0,085928616	0,098767519	-0,0128389	SOX11
cg09907720	0,033681736	0,046606773	-0,01292504	VAX2
cg06277900	0,032642901	0,045722994	-0,01308009	HSF4;HSF4;FBXL8
cg18863333	0,047946429	0,061726121	-0,01377969	SOX11
cg00731411	0,091184263	0,105028957	-0,01384469	VAX2

cg08806496	0,053258848	0,067170109	-0,01391126	NR2F1
cg08278487	0,14768025	0,161606557	-0,01392631	POU4F1
cg05241355	0,104835118	0,118790117	-0,013955	OTX2
cg08432727	0,083950091	0,097950029	-0,01399994	SOX11
cg08370996	0,078108263	0,092228709	-0,01412045	NR2F2;NR2F2
cg04641787	0,19408545	0,208278668	-0,01419322	FLJ45983;GATA3;GATA3;FLJ45983
cg05155965	0,071398208	0,085624175	-0,01422597	NR2F2;NR2F2
cg08377570	0,81874095	0,83313752	-0,01439657	LPAR6;RB1
cg14186066	0,10460586	0,119294423	-0,01468856	SIX6;SIX6
cg26348180	0,227741125	0,242496686	-0,01475556	HES1
cg02558364	0,033367459	0,048188744	-0,01482129	VAX2
cg08734740	0,101664383	0,116558821	-0,01489444	ISL1;ISL1
cg14114517	0,100329123	0,115323968	-0,01499485	LHX1;LHX1
cg20585530	0,073903242	0,089303487	-0,01540024	SIX6
cg03547745	0,037042693	0,052636005	-0,01559331	SOX9;SOX9
cg01213381	0,2091097	0,225176909	-0,01606721	OTX2
cg15941449	0,120481238	0,13655381	-0,01607257	DLX1;DLX1
cg26067250	0,109261851	0,125393638	-0,01613179	DLX1;DLX1
cg20267441	0,058691245	0,074837964	-0,01614672	VAX2
cg16185834	0,2084809	0,224719334	-0,01623843	VSX1;VSX1
cg05194810	0,127112396	0,143512384	-0,01639999	DLX1;DLX1;DLX1;DLX1
cg15272362	0,082550021	0,099049634	-0,01649961	VSX1;VSX1
cg11018337	0,031569473	0,048196638	-0,01662717	FLJ45983;GATA3;GATA3;FLJ45983
cg10791926	0,146623356	0,163498163	-0,01687481	DLX1;DLX1
cg01941895	0,135503383	0,152816973	-0,01731359	VSX1;VSX1
cg20008332	0,037702483	0,055024203	-0,01732172	SOX11
cg13826890	0,075075238	0,092613681	-0,01753844	OTX2
cg13411015	0,090871035	0,108475486	-0,01760445	OTX2

cg18815943	0,026740895	0,044396997	-0,0176561	FOXE3
cg02011639	0,095347873	0,113043486	-0,01769561	FOXN4
cg16043357	0,100235658	0,117980863	-0,01774521	VAX1;VAX1
cg18646207	0,043455862	0,061613259	-0,0181574	VAX1;VAX1
cg25682299	0,067959074	0,087329979	-0,0193709	POU4F1
cg15023858	0,840637758	0,8601289	-0,01949114	MITF;MITF
cg15219145	0,045295402	0,065312726	-0,02001732	TFAP2A;TFAP2A;TFAP2A;TFAP2A
cg05323725	0,131769681	0,15203455	-0,02026487	LHX1;LHX1
cg17412886	0,079467211	0,100058746	-0,02059153	ISL1
cg08245702	0,05077637	0,071390558	-0,02061419	VAX2
cg07411432	0,120907918	0,141786058	-0,02087814	NR2E1
cg23316987	0,155037907	0,175931919	-0,02089401	OTX2;OTX2
cg01789478	0,15635342	0,177617877	-0,02126446	NEUROD1
cg16924616	0,146658661	0,168470985	-0,02181232	DLX5
cg14834938	0,057543649	0,07950044	-0,02195679	ISL1
cg13543854	0,109520304	0,131758551	-0,02223825	FLJ45983;GATA3;GATA3;FLJ45983
cg19976235	0,281794158	0,305017737	-0,02322358	OTX2;OTX2
cg06022942	0,044719944	0,068134551	-0,02341461	FLJ45983;GATA3;GATA3;FLJ45983
cg25413843	0,447777033	0,471740134	-0,0239631	VAX2
cg12077194	0,71004135	0,734187354	-0,024146	ATOH7
cg18449120	0,185668133	0,209859381	-0,02419125	SOX11
cg01587896	0,057554817	0,082827466	-0,02527265	ISL1
cg20575761	0,169964575	0,196627571	-0,026663	OTX2
cg16318053	0,041421294	0,068278221	-0,02685693	ISL1;ISL1
cg08165221	0,076080266	0,103099099	-0,02701883	SOX11
cg07130890	0,229821733	0,257001889	-0,02718016	DLX1;DLX1
cg11732642	0,037474768	0,064868734	-0,02739397	SOX9
cg08616702	0,166703889	0,194211726	-0,02750784	NR2E1

cg22796507	0,063745498	0,091470403	-0,02772491	FOXE3
cg18451814	0,160950725	0,189177311	-0,02822659	OTX2
cg08640634	0,049590762	0,077967588	-0,02837683	NR2E1
cg21239311	0,062579391	0,090961968	-0,02838258	NR2E1
cg02631468	0,060904534	0,089605702	-0,02870117	VSX1;VSX1
cg11769960	0,113254568	0,142246377	-0,02899181	LHX1
cg07907745	0,14140789	0,170433794	-0,0290259	FLJ45983;GATA3;GATA3;FLJ45983
cg20927661	0,032673362	0,061941819	-0,02926846	SOX11;SOX11
cg26757388	0,2557499	0,285069851	-0,02931995	DLX5
cg17650274	0,176265265	0,205749594	-0,02948433	VSX1;VSX1
cg06966811	0,208219908	0,237796026	-0,02957612	OTX2
cg17076890	0,159999297	0,189906306	-0,02990701	PTF1A
cg03767531	0,051934863	0,082322073	-0,03038721	NEUROD1
cg11711420	0,120332619	0,15110774	-0,03077512	ISL1;ISL1
cg15840438	0,8158274	0,84677548	-0,03094808	PITX3
cg14909730	0,063707676	0,094671432	-0,03096376	TFAP2A;TFAP2A
cg04330371	0,055830219	0,087711751	-0,03188153	NR2F2;NR2F2;NR2F2;MIR1469;NR2F2
cg08917478	0,091073238	0,123033731	-0,03196049	ISL1
cg25603277	0,138553887	0,170711865	-0,03215798	PAX6
cg03657766	0,117379108	0,150133838	-0,03275473	DLX2
cg01281911	0,040019807	0,072804551	-0,03278474	FOXE3
cg26595643	0,035205325	0,068035708	-0,03283038	VAX1;VAX1
cg26002103	0,120272133	0,154258276	-0,03398614	DLX2
cg19762747	0,177910498	0,212259034	-0,03434854	CRX
cg11290922	0,093734255	0,129523461	-0,03578921	LHX1
cg24837370	0,169997705	0,206161985	-0,03616428	SOX11
cg16793394	0,133263417	0,169583783	-0,03632037	OTX2
cg25684999	0,056733887	0,093539855	-0,03680597	PTF1A

cg22359606	0,07419921	0,111018736	-0,03681953	NEUROD1
cg22746058	0,066566186	0,1047593	-0,03819311	PTF1A
cg16057598	0,112219538	0,150894137	-0,0386746	DLX2
cg16407139	0,736435358	0,775130903	-0,03869554	ATOH7
cg01169726	0,105365581	0,144960297	-0,03959472	DLX5
cg02086467	0,075373346	0,114983683	-0,03961034	OTX2
cg22660578	0,035301154	0,074925737	-0,03962458	LHX1
cg03950614	0,161944217	0,201750369	-0,03980615	NR2E1
cg07829379	0,102051493	0,142266992	-0,0402155	LHX1
cg20250426	0,560696117	0,600946194	-0,04025008	DLX5
cg20652607	0,092250838	0,133265193	-0,04101436	NR2E1
cg06714480	0,087471004	0,12872595	-0,04125495	NEUROD1
cg00930455	0,215023125	0,256705526	-0,0416824	DLX5
cg04535746	0,725317217	0,767103149	-0,04178593	MITF;MITF;MITF;MITF
cg18240331	0,036113929	0,078147324	-0,04203339	TFAP2A
cg02836529	0,075282742	0,117828105	-0,04254536	NEUROD1
cg18241942	0,2578095	0,300991006	-0,04318151	OTX2
cg08833577	0,050697752	0,094174757	-0,043477	VAX1;VAX1
cg01406988	0,106420198	0,150022131	-0,04360193	MEIS1;MEIS1
cg02209932	0,125536454	0,169633412	-0,04409696	MEIS1
cg17037282	0,074973044	0,119184414	-0,04421137	ISL1
cg01897496	0,08529506	0,131169517	-0,04587446	NEUROD1;NEUROD1
cg09426103	0,126400226	0,17288022	-0,04647999	DLX5
cg05721515	0,149224344	0,196256436	-0,04703209	FLJ45983;GATA3;GATA3;FLJ45983
cg22976218	0,109049538	0,156623983	-0,04757445	NEUROD1
cg09325003	0,554469633	0,602162706	-0,04769307	MITF;MITF
cg14098681	0,058622423	0,106345919	-0,0477235	FLJ45983;GATA3;GATA3;GATA3;GATA3;FLJ45983
cg19711579	0,070759998	0,118868745	-0,04810875	NEUROD1

cg17419299	0,7677266	0,815841189	-0,04811459	RB1
cg02014107	0,125572208	0,173887878	-0,04831567	DLX2
cg00263760	0,091690853	0,14018308	-0,04849223	VAX1;VAX1
cg09255586	0,172522626	0,222314659	-0,04979203	TFAP2A;TFAP2A
cg06391468	0,090748499	0,140947445	-0,05019895	SOX9;SOX9
cg15989068	0,125765663	0,176426265	-0,0506606	SOX11;SOX11
cg21410991	0,126923672	0,17982734	-0,05290367	ISL1
cg15607672	0,211694208	0,265065909	-0,0533717	OTX2
cg00906973	0,143404236	0,197241631	-0,0538374	TFAP2A
cg25739875	0,129140358	0,183208297	-0,05406794	TFAP2A;TFAP2A
cg15236866	0,278830983	0,332989066	-0,05415808	DLX1;DLX1
cg13023623	0,135276443	0,190121483	-0,05484504	OTX2
cg11063729	0,115972671	0,17179872	-0,05582605	TFAP2A;TFAP2A
cg03091551	0,092299048	0,149228533	-0,05692948	OTX2
cg07875786	0,111084518	0,170414494	-0,05932998	NR2F1
cg25721451	0,034394148	0,095217041	-0,06082289	MEIS1;MEIS1
cg02036261	0,055514868	0,117607054	-0,06209219	OTX2
cg05436064	0,030385553	0,094595169	-0,06420962	HSF4;HSF4;FBXL8
cg16226962	0,178446533	0,244441143	-0,06599461	TFAP2A;TFAP2A
cg06870728	0,216321425	0,28242842	-0,066107	FLJ45983;GATA3;GATA3;FLJ45983
cg13409449	0,062174232	0,129302377	-0,06712814	GATA3;GATA3
cg23668184	0,028671293	0,097799297	-0,069128	SOX11;SOX11
cg23271234	0,330915292	0,400457837	-0,06954255	VAX2
cg13221796	0,607176792	0,677272954	-0,07009616	RB1
cg02162880	0,117351593	0,188889675	-0,07153808	TFAP2A
cg18705279	0,095760864	0,169192846	-0,07343198	HSF4;HSF4;FBXL8
cg19015611	0,501794517	0,575714134	-0,07391962	VAX2
cg26870771	0,142178528	0,225844909	-0,08366638	HSF4;HSF4;HSF4;HSF4;FBXL8

cg03456938	0,113686019	0,198604401	-0,08491838	HSF4;FBXL8;HSF4
cg22201614	0,102143768	0,187146807	-0,08500304	SOX9
cg21318213	0,131340459	0,21870876	-0,0873683	NR2F2;NR2F2;NR2F2;MIR1469;NR2F2
cg11132582	0,743847783	0,831561154	-0,08771337	POU2F1
cg17083494	0,374468817	0,463090754	-0,08862194	DLX5
cg00405198	0,042391448	0,135539684	-0,09314824	TFAP2A
cg09030672	0,11722983	0,2105278	-0,09329797	HSF4;HSF4;FBXL8
cg26998537	0,215044536	0,308741451	-0,09369692	OTX2
cg14606431	0,301585683	0,395533051	-0,09394737	VAX2
cg18732032	0,1673429	0,262019077	-0,09467618	TFAP2A
cg14327531	0,197744267	0,294328511	-0,09658424	GATA3;GATA3
cg20434178	0,180355524	0,28089684	-0,10054132	DLX2
cg21621114	0,272717917	0,37734702	-0,1046291	CRX
cg09738906	0,174293983	0,279781794	-0,10548781	NR2F2;NR2F2;NR2F2;MIR1469;NR2F2
cg17697633	0,044983332	0,152265829	-0,1072825	TFAP2A
cg07492757	0,130129069	0,244453183	-0,11432411	TFAP2A
cg23414876	0,120610043	0,240545576	-0,11993553	HSF4;HSF4;HSF4;HSF4;FBXL8
cg17557766	0,447107025	0,572295349	-0,12518832	TFAP2A
cg04737114	0,248449442	0,374354589	-0,12590515	DLX5
cg16876790	0,26488535	0,391999237	-0,12711389	DLX5
cg23115387	0,302904067	0,430038563	-0,1271345	FOXE3
cg07816687	0,05879929	0,1859918	-0,12719251	HSF4;HSF4;FBXL8
cg19962750	0,456776075	0,58518338	-0,12840731	DLX5
cg13286614	0,396361333	0,5289366	-0,13257527	DLX5
cg24797840	0,150966839	0,284917723	-0,13395088	FLJ45983;GATA3;GATA3;FLJ45983
cg08770358	0,755127408	0,894029851	-0,13890244	RB1
cg27175287	0,3109839	0,450625271	-0,13964137	FOXE3;FOXE3
cg23365739	0,2059815	0,347877251	-0,14189575	OTX2;OTX2

cg01448276	0,208340192	0,351171306	-0,14283111	DLX5
cg03958979	0,14434855	0,289891789	-0,14554324	NR2E1
cg17916835	0,464858667	0,616178683	-0,15132002	DLX5
cg13298147	0,436129592	0,591490526	-0,15536093	TFAP2A;TFAP2A
cg17255712	0,171412523	0,330973417	-0,15956089	SOX9
cg19303187	0,306776767	0,468598	-0,16182123	DLX2
cg10243939	0,182524933	0,347853134	-0,1653282	DLX5
cg27283345	0,439165567	0,607386289	-0,16822072	TFAP2A;TFAP2A
cg07721569	0,177537471	0,351158563	-0,17362109	TFAP2A
cg06880667	0,343296392	0,529005114	-0,18570872	TFAP2A;TFAP2A
cg24228707	0,151735794	0,338273711	-0,18653792	DLX1;DLX1
cg11079583	0,318421433	0,509485174	-0,19106374	TFAP2A
cg13373589	0,325758892	0,517527103	-0,19176821	TFAP2A;TFAP2A
cg05597836	0,305402242	0,503556454	-0,19815421	DLX5
cg20540714	0,162656642	0,363667425	-0,20101078	TFAP2A;TFAP2A
cg04506585	0,123452366	0,326078526	-0,20262616	SOX9
cg14993900	0,411564183	0,6142268	-0,20266262	TFAP2A
cg23535596	0,295566742	0,505354253	-0,20978751	TFAP2A;TFAP2A
cg24902920	0,415713683	0,627676697	-0,21196301	TFAP2A;TFAP2A
cg10129408	0,238253192	0,453829811	-0,21557662	TFAP2A
cg07931844	0,343989683	0,569568157	-0,22557847	NR2E3;NR2E3
cg00689580	0,191042475	0,4216508	-0,23060833	TFAP2A
cg20806345	0,160804657	0,397822229	-0,23701757	NEUROD1
cg26235659	0,381272608	0,621805037	-0,24053243	NR2E3;NR2E3
cg14429436	0,587170808	0,840847646	-0,25367684	RB1
cg04664328	0,601671183	0,859438771	-0,25776759	NR2E3;NR2E3
cg07890954	0,388674208	0,651049366	-0,26237516	NR2E3;NR2E3
cg11350586	0,359425375	0,635134043	-0,27570867	SOX9

cg04438997	0,31780245	0,595862063	-0,27805961	SOX9
cg16586105	0,595471642	0,897736674	-0,30226503	NR2E3;NR2E3
cg06945747	0,329999383	0,639977294	-0,30997791	CRX
cg10899301	0,198699433	0,510919903	-0,31222047	TFAP2A;TFAP2A
cg15151364	0,242213892	0,5763779	-0,33416401	POU2F1
cg05175964	0,40620755	0,784241063	-0,37803351	NR2E3;NR2E3
cg13089335	0,289018258	0,698625086	-0,40960683	POU2F1

Supplementary Table S4. List of genes showing differential methylation (p-value <0.5) between neuroretina from healthy donor and background non-proliferative diabetic retinopathy (NPDR).

p-value	*ID*	*Location*	*Type(s)*	*Drug(s)*
8,23E09	DNAJB1	Nucleus	other	
8,23E09	KATNB1	Cytoplasm	enzyme	
2,47E-03	SERPINB8			
1,56E-03	ACOT2	Cytoplasm	enzyme	
1,56E-03	CROCC	Plasma Membrane	other	
1,56E-03	FNDC3A	Cytoplasm	other	
1,56E-03	GRIK3	Plasma Membrane	ion channel	
1,56E-03	MAP3K1	Cytoplasm	kinase	
1,56E-03	NLK	Nucleus	kinase	
1,56E-03	NUCB1	Cytoplasm	other	
1,56E-03	PASK	Cytoplasm	kinase	
1,56E-03	PRKACA	Cytoplasm	kinase	
1,56E-03	PTH1R	Plasma Membrane		PTH
1,56E-03	RNF44	Other	transcription	
1,56E-03	SOX4	Nucleus		
1,56E-03	STK32B	Other	kinase	
1,56E-03	TFR2	Plasma Membrane	transporter	
1,56E-03	TMEM161A	Other	other	
1,56E-03	TRIM36	Cytoplasm	other	
1,56E-03	ZFAND2A	Other	other	
1,56E-03	HMGCR		enzyme	
1,56E-03	CLASP1	Cytoplasm	other	
1,56E-03	FAU;MRPL49			
1,56E-03	RILPL2			
1,56E-03	NTM;NTM			
1,56E-03	SLC26A9			
1,56E-03	ASAP3			
1,22E-03	IFI30	Cytoplasm	enzyme	
1,09E-03	EXO1	Nucleus	enzyme	
9,87E-04	ARAP1	Cytoplasm	other	
9,87E-04	CDK6	Nucleus	kinase	palbociclib, flavopiridol LEE011
9,87E-04	CHAT	Nucleus	enzyme	
9,87E-04	DPP10	Extracellular Space	translation	
9,87E-04	EIF2B2	Cytoplasm	regulator	
9,87E-04	FAM195A	Other	other	
9,87E-04	GPHN	Plasma Membrane	enzyme	
9,87E-04	NHLRC4	Other	other	
9,87E-04	SLC39A14	Plasma Membrane	transporter	
9,87E-04	C11Orf60			
9,84E-04	CTNNBL1			
5,76E-04	C4orf49	Cytoplasm	other	
5,76E-04	MLEC	Plasma Membrane	other	
3,29E-04	ADAP2	Cytoplasm	other	
3,29E-04	WNT3	Extracellular Space	other	
1,65E-04	CDC42EP3	Cytoplasm	other	
1,65E-04	FUT4	Cytoplasm	enzyme	

Supplementary Table S5. Primer sequences for bisulfite sequencing, Methylation – Specific PCR (MSP), RT-PCR and quantitative RT-PCR (qPCR) reactions.

Bisulfite sequencing

Gene	*EntrezID*	*5´primer*	*3´primer*
EXOC2	55770	ttgggttgttgtagagtgatag	aatcacaacactcaacaatacc
GUCA1B	2979	ggtttttaaagtgttgggatt	aaaacactccatcacaaacttc
ITGA7	3679	gggatttgttatttttttgg	aacaaaaaaataaaaaaaccca
KCNB1	3745	gaggtgtgygatgattatagtt	tccaataaatcccaaaattttt
LRIT1	26103	aagtgggtagttgttgagtttg	caacccaacctaactaacaaaa
MYT1	4661	ttggagttttaagggtttatgg	tttcctacaaacaaaaccttca
NDRG2	57447	aaggttatattgggagtggtag	aatcaaaattctcactccttct
PAX6 (1)	5080	tttttgaatgggtttttattg	acttcataaaccctaacccaat
PAX6 (2)	5080	taagtgttttggtttttttgg	aaatttctctaccrcaacttc
PDE6A	5145	agattggatttgttgtagattg	caaaaaaaaacacaacttcttc
PITX2	5308	agggattaattttgtgattttattg	aaaactccctaaaccccttc
RAX	30062	gatataaatggttttggggtg	ctaaccccctctaactcacct
SIX6	4990	ggttttagggttttttttttttt	aaatccatccctaaacaaaaat
VSX2	338917	gggaattttttgggttttag	ccttattcaaacccaaaatctc

Methylation- Specific PCR (MSP)

Gene	*Entrez ID*	*5´primer*	*3´primer*
ITGA7	3679		
unmethylated		ggtgttttgtgtaaggagggt	aacaaatacaataactcaccaactc
methylated		gttttgcgtaaggagggc	acgaatacgataactcaccaac
NDRG2	57447		
unmethylated		ggtagagttgggatttgaattt	tcctctctaaactcaaaccaaaa
methylated		agagtcgggattcgaattc	tcctctctaaactcgaaccg
PITX2	5308		
unmethylated		agagtttttgtttgtgttttt	cctacatctcaacctctcctaaa
methylated		gttttcgttcgcgttttc	acgtctcgacctctcctaaa
RAB31	11031		
unmethylated		aggaatgttgagaaaggtaggtt	cataacaacacataaaaatctac
methylated		aacgtcgagaaaggtaggtc	cgtaacaacgcgtaaaaatc

RT-PCR

Gene	*Entrez ID*	*5´primer*	*3´primer*
α-SMA	59	cgtggctattccttcgttact	cccctgataggacattgttag
FSP1	6275	ggcaaagagggtgacaagtt	gggctgcttatctgggaag
RPLP0	6175	caacgggtacaaacgagt	cttccttggcttcaaccttag
VIM	7431	gcaggctcagattcaggaac	gtgagggactgcacctgtct

qPCR

Gene	*Entrez ID*	*5´primer*	*3´primer*
EXOC2	55770	accggcatctctccaaatga	gcctctgcccttacctatga
MYT1	4661	ccgaggtgagctgttaaatca	cgagctcgcttgtcttcatt
PAX6	5080	cgtccatctttgcttgggaa	tctgcccgttcaacatcctt
RAX	30062	gagtacgaagcccctcgac	gctgttcctcctctgacagt
SIX6	4990	ggtgggcaactggttcaaaa	tgctggatagactggcgg
VSX2	338917	gtccacttgcagccattgg	aaagattgtcctgtgtcgcc
